# Lignin-Derivative Ionic Liquids as Corrosion Inhibitors

**DOI:** 10.3390/molecules28145568

**Published:** 2023-07-21

**Authors:** Sharon Monaci, Daniela Minudri, Lorenzo Guazzelli, Andrea Mezzetta, David Mecerreyes, Maria Forsyth, Anthony Somers

**Affiliations:** 1 POLYMAT, University of the Basque Country UPV/EHU, 20018 Donostia-San Sebastian, Spain; smonaci@deakin.edu.au (S.M.); daniela.minudri@polymat.eu (D.M.); david.mecerreyes@ehu.es (D.M.); maria.forsyth@deakin.edu.au (M.F.); 2 Institute for Frontier Materials, Deakin University, Burwood, VIC 3125, Australia; 3 Dipartimento di Farmacia, Università di Pisa, Via Bonanno Pisano 33, 56126 Pisa, Italy; lorenzo.guazzelli@unipi.it (L.G.); andrea.mezzetta@unipi.it (A.M.); 4 Ikerbasque, Basque Foundation for Science, 48009 Bilbao, Spain

**Keywords:** biobased, corrosion inhibitors, ionic liquids

## Abstract

Corrosion is a significant problem that negatively affects a wide range of structures and buildings, resulting in their premature failure, which causes safety hazards and significant economic loss. For this reason, various approaches have been developed to prevent or minimize the effects of corrosion, including corrosion inhibitors. Recently, biobased inhibitors have gained a certain interest thanks to their unique properties, eco-friendliness, and availability. Among all the green precursors, lignin is of particular interest, being a natural polymer that can be obtained from different sources including agricultural residues. Corrosion inhibitors based on ionic liquids (ILs) also present interesting advantages, such as low volatility and high tunability. If combined, it may be possible to obtain new lignin-based ILs that present interesting corrosion inhibitor properties. In this work, the inhibition properties of new biobased lignin ILs and the influence of anions and cations on the corrosion of mild steel in an aqueous solution of 0.01 M NaCl were investigated by Potentiostatic Electrochemical Impedance Spectroscopy (PEIS) and Cyclic Potentiodynamic Polarization (CPP). Moreover, the surface was characterized using SEM, EDS, and optical profilometry. The IL choline syringate showed promising performance, reducing the corrosion current after 24 h immersion in 0.01 M sodium chloride, from 1.66 µA/cm^2^ for the control to 0.066 µA/cm^2^ with 10 mM of the IL present. In addition to its performance as a corrosion inhibitor, both components of this IL also meet or exceed the current additional desired properties of such compounds, being readily available, and well tolerated in organisms and the environment.

## 1. Introduction

Corrosion is a natural process that takes place whenever a metal is exposed to the environment, leading to its oxidation and degradation over time. Due to both economic and safety concerns caused by corrosion, there are several strategies that can be adopted to mitigate the problem [1]. Among these strategies, corrosion inhibitors are one of the easiest and most efficient ways to guarantee good protection of a metal surface, such as mild steel, from corrosion [2]. Due to increasing awareness of the environmental problems linked to the use of chromate-based inhibitors, the so-called “green corrosion inhibitors”, a class of organic compounds derived from natural sources, have emerged as an interesting approach to the problem of finding effective corrosion inhibitors that are non-toxic and environmentally friendly [3]. Unlike traditional inhibitors, green corrosion inhibitors are derived from natural sources including plant extracts, biomass, and agricultural industry waste. 

Plant extracts, natural polymers, and ionic liquids are among the most common sources of green inhibitors that have received significant research attention in recent years [4]. Plant extracts present the great advantage of deriving from a natural source through an easy and low-cost extraction, and often can be recuperated from agricultural or agro- wastes [3]. On the other hand, ionic liquids offer attractive properties such as easy synthesis and negligible vapor pressure, which allow them to be recognized as a greener and safer alternative to volatile organic solvents [5]. In the context of environmental sustainability, the synthesis of ionic liquids from building blocks of natural origin is an imperative demand. During the last few years, a large number of natural compounds derived from sugars have been used for the preparation of ILs [6]. In contrast, natural products derived from natural polymers such as lignin remain an underexploited option. Therefore, the preparation of lignin-based ILs represents an interesting challenge that opens up new possibilities for developing next-generation ILs. Plant-derived polyphenols possess the ability to donate hydrogen atoms, which allows them to scavenge free radicals and inhibit oxidative stress, and for this reason, they have been recently investigated in the synthesis of natural deep eutectics solvents as corrosion protectors [7]. It is known from the literature that molecules containing unshared electron pairs and double bonds can mitigate corrosion by forming a protective layer on the metal’s surface [8,9]. Based on structurally related carboxylic derivatives of lignin, such as gallic, syringic, and vanillic acids, three different types of ILs, two aprotic and one protic, were selected for their promising structure, suitable for anticorrosion testing, and were prepared to be tested as potential corrosion inhibitors.

Amine-based compounds are widely used in the fields of corrosion protection [10]. For this reason, a very common imidazole-type cation and the biobased choline cation were selected for the two classes of aprotic ILs. Fully biobased ionic liquids can be obtained with the latter combination. Choline-based ionic liquids with gallate, syringate, and vanillate anions were previously prepared and used, mainly as hydrotropes [11,12]. However, to the best of our knowledge, these compounds were not used as antioxidant additives to date. For the case of protic ILs, the well-studied DBU cation was selected for their preparation.

While a few studies have explored the possible use of gallic acid as a corrosion inhibitor [13,14], syringic acid and vanillic acid, as far as we know, have never been investigated as potential corrosion inhibitors.

**Figure 1:**
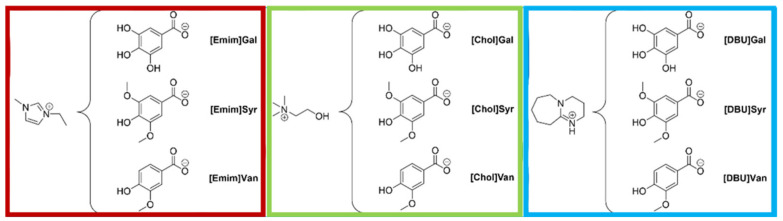
Three different families of lignin-based ILs studied as potential corrosion inhibitors.

In this work, the three aforementioned lignin derivative acids were incorporated into different ILs, as shown in Figure 1. These new ILs present the advantage of being halogen and acid-free, having tunable solubility, and the ability to suppress condensation reactions. Furthermore, the synthetic methods applied were based on previously optimized procedures aimed at reducing their environmental footprint. 

This portfolio of prepared ILs allows a comprehensive insight into the contribution of cations and diverse anions to the corrosion inhibition properties of these structurally related IL families. All compounds were characterized by ^1^H and ^13^C NMR and their thermal properties were investigated. The anticorrosion properties were electrochemically investigated in an aqueous solution of sodium chloride using mild steel as a working electrode. In order to have a more comprehensive understanding of their inhibition effects, the surfaces of mild steel after 24 h of immersion were studied by SEM, EDS, and optical profilometry.

## 2. Results and Discussion

### 2.1. Thermal Characterization

The thermal properties of the synthesized ILs were evaluated in terms of thermal stability and thermal behavior. To investigate the short-term thermal stability, thermogravimetric analysis (TGA) was carried out. This technique has been widely used to compare the thermal stability of ILs, as it permits the acquisition of data in a short time. Based on the thermogravimetric data of 66 ILs, Cao and Mu [15] identified five different levels of thermal stability, ranging from the least stable to the most stable. The lignin-based ILs were analyzed under the same conditions and the results are shown in Table 1, with the TG curves in the Supplementary Materials. Three different temperatures were identified for each IL, *T*_start_, *T*_onset_, and *T*_peak_.

**Figure 2:**
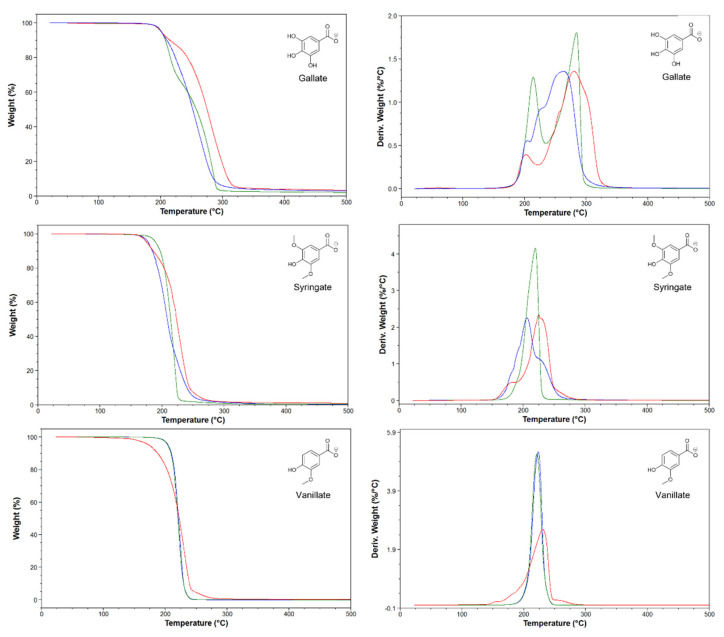
Thermal gravimetric curves (**left side**) and derivatives (**right side**) of lignin-based ILs. [Emim] (red lines), [Chol] (blue lines), and [DBU] (green lines).

The analysis of the *T*_onsets_ shows that all the ILs prepared belong to the least stable class. This result is in line with what was reported for the majority of ILs with a carboxylate anion [15]. Comparing ILs with the same anion, no evident differences in stability are observed by varying the cationic portion. The sole exception is represented by [Emim]Van, which showed a significantly lower *T*_start_ within the same series. Considering instead the *T*_start_ of ILs with different types of anions, keeping the cation unchanged, a slightly lower stability of syringate-based ILs is observed compared to gallate and vanillate, again with the exception of [Emim]Van. Further analysis of the degradation profiles shows a marked difference between the gallate-based ILs compared to syringate and vanillate (Figure 2). The gallate-based ILs are in fact the only ones that show two distinct degradation events, with the cation that seems to play a prominent role in promoting different degradation mechanisms of the ILs. For example, in the case of the choline cation, even a third degradation event is noticeable between the two main ones. However, further studies are needed to confirm and clarify the degradation mechanism of these ILs.

Differential scanning calorimetry (DSC) was used to evaluate the thermal behavior of lignin-based ILs, and the data are reported in Table 1 (thermographs are available in Supplementary Materials, Figures S23–S31). Apart from [Emim]Van, all compounds were found to be solid at room temperature. A decrease in the melting point in the order [Emim]Gal > [Emim]Syr > [Emim]Van was observed in the series of ILs with the [Emim] cation. In fact, [Emim]Gal decomposes before melting, [Emim]Syr melts at 148.0 °C, while [Emim]Van is liquid at room temperature and only displays a glass transition at −1.17 °C. The reverse trend of melting decrease is observed for the series with the [Chol] cation, namely, [Chol]Van > [Chol]Syr > [Chol]Gal. In this case, [Chol]Van melts close to the degradation temperature while [Chol]Syr and [Chol]Gal melt at 157.4 and 135.8 °C, respectively. It is also worth stressing that [Chol]Syr and [Chol]Gal showed the tendency to form undercooled liquids. In fact, a glass transition at 18.63 and 22.29 °C was observed for [Chol]Syr and [Chol]Gal, respectively, during successive heating/cooling cycles. Finally, none of the ILs of the series with the [DBU] cation melt before degrading. Only for [DBU]Gal, a solid–solid transition was observed at 76.37 °C.

### 2.2. Electrochemical Characterization

Every inhibitor was electrochemically tested through PEIS and CPP at a concentration of 5 mM to evaluate their anti-corrosion properties at a low concentration. From these preliminary results, some were selected for testing at a higher concentration (10 ppm) and immersed corrosion samples were further characterized through surface analysis. As is shown in Figure S1, the inhibitors containing vanillate as an anion, and DBU as a cation demonstrated very low impedance and consequently poor protection against corrosion. For this reason, these inhibitors were not further investigated, thus leaving 4 ILs to be further investigated.

#### 2.2.1. Potentiostatic Electrochemical Impedance Spectroscopy (PEIS)

**Figure 3:**
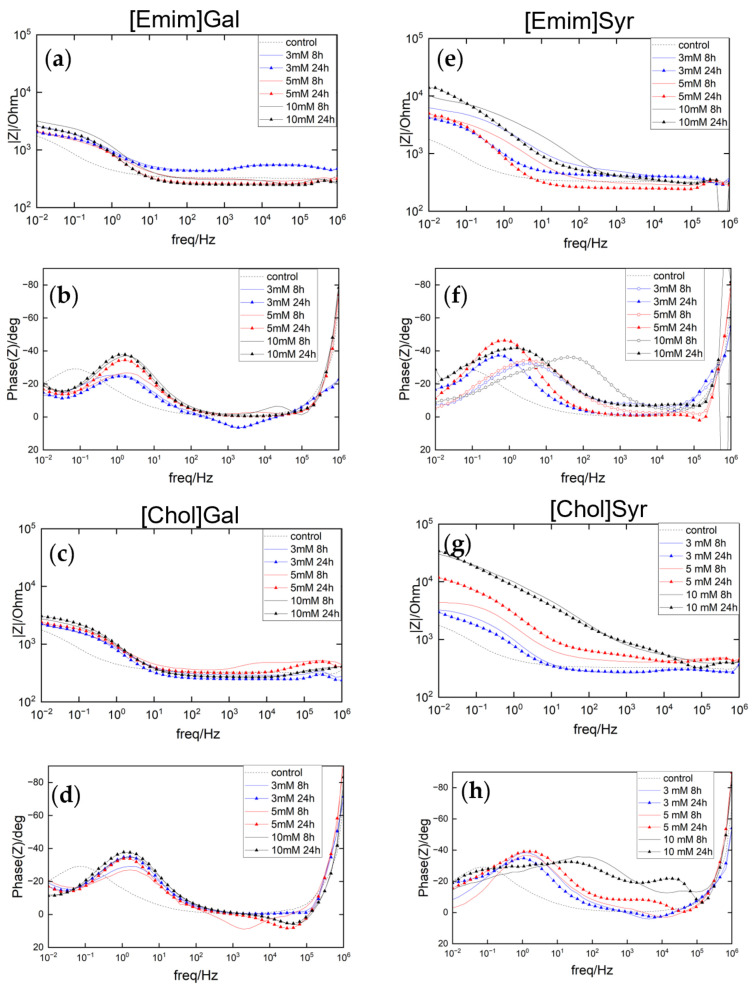
Electrochemical Impedance results of AS1020 mild steel immersed in control (0.01 M NaCl; dotted line) and inhibited solutions (0.01 M NaCl + inhibitor) of [Emim]Gal, [Emim]Syr, [Chol]Gal, [Chol]Syr 3, 5, and 10 mM (blue, red, and black lines) up to 24 h: impedance modulus (**a**,**c**,**e**,**g**) and phase angle plots (**b**,**d**,**f**,**h**).

The impedance spectra of the samples exposed to a 0.01 M NaCl aqueous solution for 24 h with and without inhibitor were measured by PEIS, with Bode and Phase angle plots shown in Figure 3. After 24 h of immersion, the mild steel control presents an impedance of 1760 Ω and a peak angle of −30° in the lower frequency range (10^−1^–10^1^ Hz). For the solutions containing the inhibitors at concentrations of 3, 5, and 10 mM, the plots for the Emim ILs show that the gallate does not seem to significantly improve the impedance, even at higher concentrations, while on the other hand with the syringate, there is an increase in the impedance with concentration. [Emim]Syr at 10 mM causes an increase in the total impedance of almost one order of magnitude (from 1700 to 14,000 Ω) with respect to the control. Regarding the phase angle, there is a shift toward more positive frequencies with the gallate anion, and the increase in the concentration causes an increase in the angle up to 40° for a concentration of 10 mM. With the syringate anion, in the phase angle data, it is still possible to observe that an increase in the concentration leads to both a shift toward more positive frequencies and to an increment of the phase angle up to 40°, which can be correlated with the formation of the inhibitor film.

For the inhibitors containing choline as a cation, the effect of the gallate and syringate anions are similar to that for the Emim, but, in this case, the syringate anion causes an increase in the impedance, from 1700 Ω for the control to 34,000 Ω with 10 mM [Chol]Syr, with it displaying an almost straight line until high frequencies. When gallate is used with choline or Emim, both the impedance spectra show little variation with concentration. 

Interestingly, the Phase angle of [Chol]Syr shows an additional peak at higher frequencies, meaning that probably there are two electrochemical processes happening on the surface of the steel. The two processes can be attributed to the corrosion process for the higher peak and to the inhibitor film for the peak at higher frequencies. This is consistent with the literature [16], where it is suggested that the shift toward more high frequencies in the phase angle of dielectric films is caused by their small time constant.

#### 2.2.2. Cyclic Potentiodynamic Polarization (CPP)

**Figure 4:**
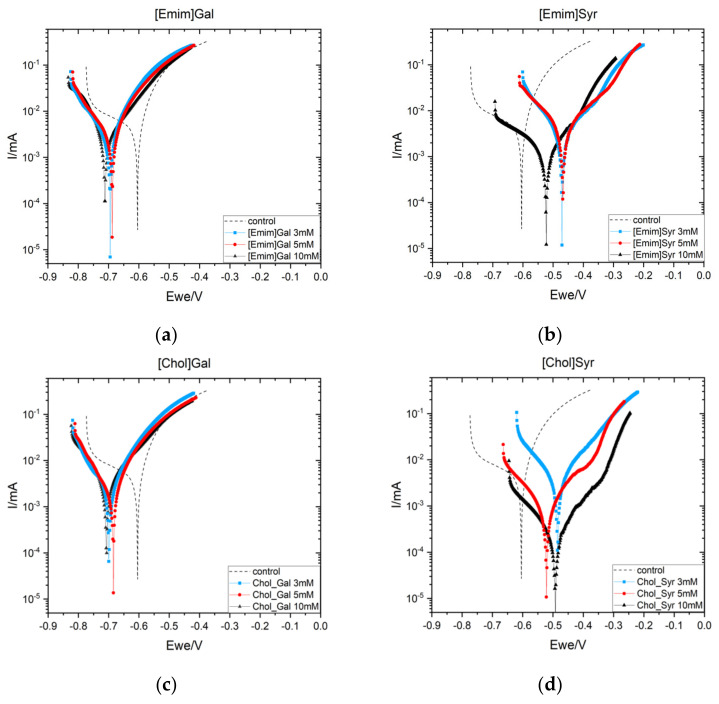
Cyclic Potentiodynamic Polarization (CPP) results of AS1020 mild steel after 24 h at OCV in control (0.01 M NaCl; dotted line) and inhibited solutions (0.01 M NaCl + inhibitor) containing [Emim]Gal 3, 5, and 10 mM (**a**) and [Emim]Syr 3, 5, and 10 mM (**b**). [Chol]Gal 3, 5, and 10 mM (**c**) and [Chol]Syr (**d**) 3, 5, and 10 mM.

To further investigate the inhibitor behavior of the ILs, Cyclic Potentiodynamic Polarization scans of AS1020 mild steel samples after 24 h of exposure in the control solution (0.01 M NaCl) and inhibited solution (0.01 M NaCl + inhibitor at different concentrations) were performed and the resulting curves are shown in Figure 4. Corrosion potential (E_corr_), corrosion current density (*i*_corr_), and inhibitor efficiency calculated using Tafel extrapolation are reported in Table 2.

The two lignin anions show distinct behaviors; when steel is immersed in the solution containing gallate, the E_corr_ presents a shift toward more negative potential with respect to the control (dotted line), while with the syringate, there is a shift toward more positive potential. A small decrease in the cathodic branch can be observed for [Emim]Gal and [Chol]Gal, with a decrease in the corrosion current significantly smaller than for [Emim]Syr and [Chol]Syr at 10 mM.

At a concentration of 10 mM, [Emim]Syr presents a shift toward less anodic potential with respect to the concentrations of 3 and 5 mM, with a significant decrease in the anodic branch and in the corrosion current, which decrease from 1.457 to 0.312 µA/cm^2^ with respect to the control. On the other hand, [Chol]Syr shows the greatest reduction in the corrosion current (from 1.457 to 0.066 µA/cm^2^) and the highest efficiency at 10 mM.

From these plots, it seems that the syringate anion leads to an inhibitor with more of an anodic effect and presents the highest inhibitor efficiency at 10 mM. 

### 2.3. Surface Characterization

Since the concentration of 10 mM showed the greatest reduction in corrosion rate from the electrochemistry, the surfaces were characterized at this concentration. 

**Figure 5:**
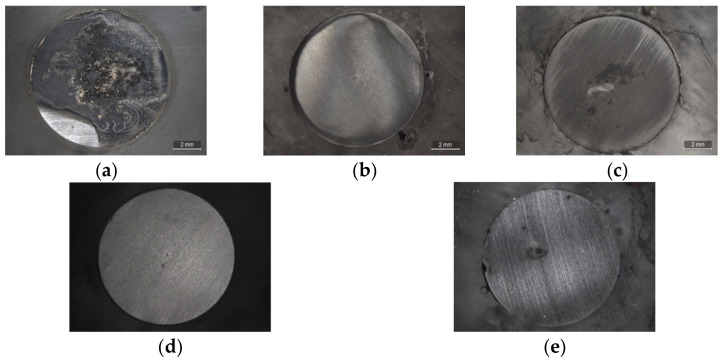
Microscope images of mild steel samples immersed in the control (**a**) and inhibited solutions containing 10 mM of [Emim]Gal (**b**), [Emim]Syr (**c**), [Chol]Gal (**d**), [Chol]Syr (**e**).

**Figure 6:**
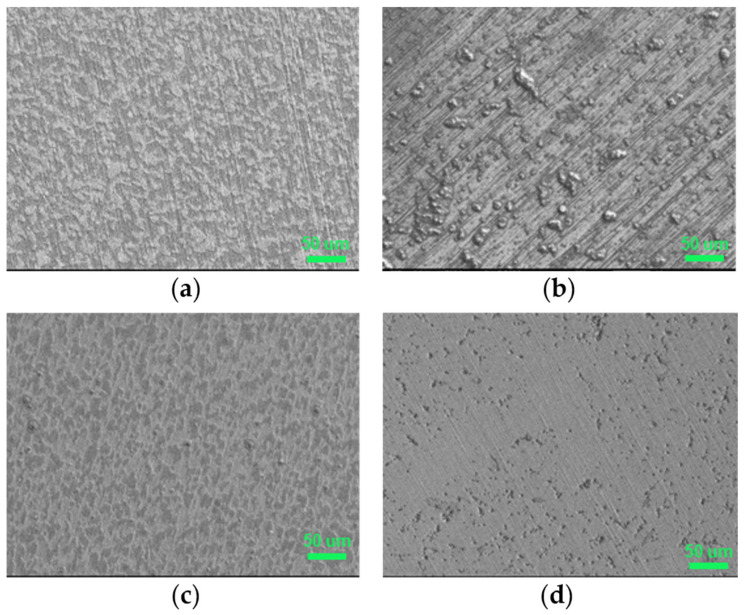
SEM micrographs of mild steel samples immersed in the inhibited solutions containing 10 mM of [Emim]Gal (**a**), [Emim]Syr (**b**), [Chol]Gal (**c**), [Chol]Syr (**d**).

The surface of AS1020 mild steel after 24 h of immersion in control and inhibited solutions were studied by optical microscopy, scanning electron microscopy, and optical profilometer. In Figure 5 and Figure 6, optical images and SEM micrographs show how a different anion can affect the surface of the sample. In more detail, the optical microscope images in Figure 5 show a uniformly corroded surface in the case of [Emim]Gal and [Chol]Gal, while is possible to see some pits in [Emim]Syr and [Chol]Syr. 

The microscope images are consistent with the CPP plots, where the presence of a few pits in the sample immersed in solutions containing syringate as an anion and uniform corrosion in the sample immersed in solutions containing gallate as an anion confirm the anodic and cathodic behavior of the respective ILs.

From the SEM, both the surfaces of [Emim]Gal and of [Chol]Gal appear to be clean, without any deep pits or deposits of corrosion products, but uniformly etched. It is known from the literature that Gallic acid forms binary and ternary complexes with iron (III) in aqueous solutions, which leads to the formation of a solid product [17]. Since the surface is clean, it seems that this complex does not provide any type of protection. 

The EDS in Figure S2 shows that the surface of [Emim]Gal is mainly characterized by iron and oxygen with a small percentage of carbon (not higher than the percentage of carbon found in the control), while the surface of [Chol]Gal in Figure S3 shows the same kind of corrosion, with some deposit on the surface that could be attributed to the cation choline from the higher percentage of carbon. In the case of [Emim]Gal, the EDS shows no sign of nitrogen and a minor presence of oxygen and carbon. The carbon content is found to be even lower than the one found for pristine mild steel (Figure S2). Based on this finding, it can be asserted that the inhibitor is not interacting with the surface to form a protective layer, but, most likely, the gallate anion participates in the formation of the aforementioned complex with iron, which remains soluble in the solution [17]. Furthermore, the limited percentages of carbon and oxygen are insufficient to confirm the presence of the [Emim] cation on the surface, leading to the conclusion that this ion also exhibits weak interactions with the metal surface and, therefore, does not contribute to its protection. The use of [Chol] instead of [Emim] as a cation does not influence the aspect of the surface as dramatically as the anion, but it seems that choline better interacts with the steel surface and inhibits the corrosion process, in accordance with electrochemistry. 

The use of syringate instead of gallate for both Emim and choline leads to the formation of deposits on the surface, which are smaller for [Chol]Syr than for [Emim]Syr, meaning the inhibitor [Chol]Syr may shut down the corrosion reaction faster. The EDS in Figure S4 for [Emim]Syr shows a high percentage of carbon on the surface, which can be attributed to the inhibitor. In the case of [Chol]Syr, the surface was clean for most of the sample, but with some pits. For this reason, four different areas were analyzed by EDS in Figure S5 to better understand the nature of the deposits. Area number 1 is used as reference, since it appears to be clean with no evident signs of corrosion. Area numbers 2 and 3 present a significant decrease in the iron percentage and an increase in the carbon amount (36% and 45% respectively). Every area shows the presence of nitrogen, especially areas 2 (5.3%) and 3 (4.2%): the presence of carbon and nitrogen can be considered clear proof of the presence of the inhibitor and confirms the presence of the choline cation that contains nitrogen in its structure. Moreover, the percentage of oxygen goes from 1% in area 1 to 24.3% and 25.6% in areas 2 and 3, respectively. This increase may be due to two reasons: the presence of iron oxides as corrosion products, not supported by the decrease in the iron percentage, or the presence of the inhibitor (characterized by hydroxyl groups and a carboxylic moiety) on the surface of the metal, forming a protective precipitate on the surface. 

According to the electrochemistry and SEM micrographs, the roughness of the surface of the samples calculated through optical profilometry and reported in Table S1 showed that [Chol]Syr was able to inhibit the corrosion process and protect the surface during the 24 h of immersion, resulting in an overall roughness close to the pristine steel (S_a_ of 0.109 μm for [Chol]Syr vs. 0.101 μm for control). [Chol]Gal presented quite a smooth surface, with an S_a_ of 0.161 μm, while [Emim]Syr and [Emim]Gal presented an S_a_ of 0.241 and 0.273, respectively.

## 3. Materials and Methods

### 3.1. Materials

1,8-Diazabicyclo [5.4.0]undec-7-ene 99% (DBU) was obtained from Alfa Aesar, Thermo Fisher, Waltham, MA, USA. Choline hydroxide 98% (ChOH), gallic acid >98.0%, vanillic acid >98.0%, and syringic acid >97.0% were purchased by TCI Chemicals. 1-Ethyl-3-methylimidazolium methylcarbonate [Emim MeCO_3_] (98%) methanol solutions were obtained from Proionic GmbH, Grambach, Austria. Mild Steel AS1020, NaCl (>99.5% w/t), and MiliQ water were purchased from Sigma-Aldrich, St. Louis, MI, USA (Merck Life Science). All the employed reagents and solvents were used without further purification where not differently mentioned.

### 3.2. Synthetic Pathways

The three different classes of lignin-derived ILs were prepared using methods previously optimized for similar compounds.

#### 3.2.1. General Procedure for the Synthesis of Imidazolium Lignin-Based ILs

**Figure 7:**
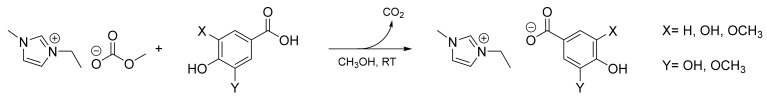
Synthetic steps for [Emim]Gal; [Emim]Syr; [Emim]Van.

The imidazolium-based ILs were synthesized from a methyl carbonate precursor in accordance with the procedure developed for carboxylated ILs by Mero et al. [18]. The proper phenolic acid (1 equiv.) was added to a commercially available methanolic solution of the methyl carbonate IL (15.77 g of methanol solution, 5 g of pure [Emim]MeCO_3_). The concentration of methylcarbonate IL in methanol solution was previously determined by volumetric titration under stirring using a standard 0.1 M HCl solution (Eutech pH meter, pH 7.00, calibrated with three standard buffer solutions at pH 4.01, 7.00, and 10.00). The resulting mixture was stirred for 2 h at room temperature and the reaction solvent was evaporated under reduced pressure (15 mbar) at 45 °C for 3 h to recover the target compounds in quantitative yield. The structure of the compounds was confirmed by ^1^H and ^13^C-NMR and the spectra are reported in the Supplementary Materials. The synthetic steps of the synthesis are reported in Figure 7. 

##### 3-Ethyl-1-methyl-1H-imidazol-3 ium Gallate ([Emim]Gal)

The preparation of [Emim]Gal (99% yield, light yellow solid), whose synthetic pathway is shown in Figure 7, was performed according to the general procedure for imidazolium-based ILs. ^1^H-NMR (400 MHz, MeOD) δ (ppm) s 8.78 (s, 1H, C(2)*H* imidazolium), 7.50 and 7.43 (2d, 2H, C(4-5)*H* imidazolium), 6.98 (s, 2H, 2X C(orto)*H* gallic), 4.15 (q, 2H, NC*H*_2_CH_3_ chain), 3.83 (s, 3H, NC*H*_3_), 1.45 (t, 3H, NCH_2_C*H*_3_); ^13^C-NMR (100 MHz, MeOD) δ (ppm) 175.6 (*C*OO^−^, gallic), 146.1 (C-3 and C-5, gallic), 137.4 (C-4, gallic), 136.8 (C-2 imidaz), 129.6 (C-1, gallic), 124.8, 123.1 (C-4 and C-5, imidaz), 110.0 (C-2 and C-6, gallic), 45.94 (N*C*H_2_ CH_3_), 36.36 (N*C*H_3_), 15.44 (NCH_2_ *C*H_3_). 

##### 3-Ethyl-1-methyl-1H-imidazol-3-ium Syringate ([Emim]Syr)

The preparation of [Emim]Syr (99% yield, brown solid) was performed according to the general procedure for imidazolium-based ILs. ^1^H-NMR (400 MHz, MeOD) δ (ppm) s 8.92 (s, 1H, C(2)*H* imidazolium), 7.63 and 7.55 (2d, 2H, C(4-5)*H* imidazolium), 7.34 (s, 2H, 2× C(orto)*H* syring), 4.24 (q, 2H, NC*H*_2_CH_3_ chain), 3.92 (s, 3H, NC*H*_3_), 3.89 (s, 6H, 2×(OC*H*_3_)), 1.53 (t, 3H, NCH_2_C*H*_3_); ^13^C-NMR (100 MHz, MeOD) δ (ppm) 175.3 (*C*OO^−^, syring), 148.7 (C-3 and C-5, syring), 140.23 (C-4, syring), 137.7 (C-2 imidaz), 128.9 (C-1, gallic), 124.9, 123.3 (C-4 and C-5, imidaz), 108.2 (C-2 and C-6, syring), 56.7 (2×(O*C*H_3_), syring), 45.99 (N*C*H_2_ CH_3_), 36.38 (N*C*H_3_), 15.52 (NCH_2_ *C*H_3_).

##### 3-Ethyl-1-methyl-1H-imidazol-3-ium Vanillate ([Emim]Van)

The preparation of [Emim]Van (99% yield, brown liquid) was performed according to the general procedure for imidazolium-based ILs. ^1^H-NMR (400 MHz, MeOD) δ (ppm) s 8.91 (s, 1H, C(2)*H* imidazolium), 7.60 and 7.53 (2d, 2H, C(4-5)*H* imidazolium), 7.59 (d, 1H, C(2)*H* van), 7.49 (dd, 1H, C(6)*H* van), 6.76 (d, 1H, C(5)*H*), 4.23 (q, 2H, NC*H*_2_CH_3_ chain), 3.90 (s, 3H, NC*H*_3_), 3.89 (s, 3H, OC*H*_3_), 1.51 (t, 3H, NCH_2_C*H*_3_); ^13^C-NMR (100 MHz, MeOD) δ (ppm) 175.4 (*C*OO^−^, van), 151.2 (C-4, van), 148.4 (C-3, van), 137.4 (C-2 imidaz), 130.1 (C-1, van), 124.8, 123.2 (C-4 and C-5, imidaz), 124.2 (C-6, van), 115.5 (C-5, van), 114.1 (C-2, van), 56.3 (O*C*H_3_), syring), 45.94 (N*C*H_2_ CH_3_), 36.35 (N*C*H_3_), 15.49 (NCH_2_ *C*H_3_).

#### 3.2.2. General Procedure for the Synthesis of Cholinium Lignin-Based ILs

**Figure 8:**
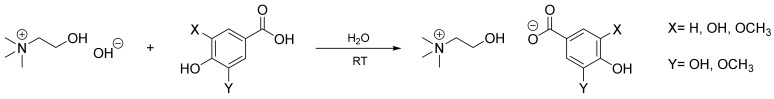
Synthetic steps for Cholinum gallate [Chol]Gal; [Chol]Syr; [Chol]Van..

Choline-based ILs were prepared from a commercially available aqueous solution of choline hydroxide (ChOH). Choline hydroxide (ChOH) solution was titrated with aqueous HCl 1 M (NIST Standard Solution, ready to use, for volumetric analysis, Fisher Chemical™), giving the exact percentage of ionic liquid in water (47.79 wt%). An equimolar amount of the proper phenolic acid was added to the titrated solution and the mixture stirred for 1 h. Finally, the solvent was removed under a vacuum (10 mbar) at 60 °C for 8 h to obtain the corresponding reaction products, all of which were light yellow. The structure of the compounds was checked by ^1^H and ^13^C-NMR and the spectra, reported in the Supplementary Materials, agreed with those reported in the literature [19]. The synthetic steps of the synthesis are reported in Figure 8.

The preparation of [Chol]Gal (99% yield, light yellow solid) was performed according to the general procedure for imidazolium-based ILs. ^1^H NMR (400 MHz, MeOD): δ 7.05 (s, 2H, H-2 e H-6), 4.04 − 3.99 (m, 2H, NC*H*_2_CH_2_OH), 3.50 − 3.46 (m, 2H, NCH_2_C*H*_2_OH), 3.20 (s, 9H, N(C*H*_3_)_3_)). ^13^C NMR (100 MHz, MeOD) δ 175.4 (s, COO), 145.6 (C-3 e C-5), 136.9 (C-4), 129.5 (C-1), 110.2 (C-2 e C-6), 68.91 (N*C*H_2_CH_2_OH), 56.89 (NCH_2_*C*H_2_OH), 54.91, 54.87, 54.84 (N(*C*H_3_)_3_). ^1^H NMR and ^13^C NMR data agree with those reported in the literature [12].

##### Trimethyl-β-hydroxyethyl-ammonium Syringate ([Chol]Syr) 

The preparation of [Chol]Syr (99% yield, light yellow solid) was performed according to the general procedure for imidazolium-based ILs. ^1^H NMR (400 MHz, MeOD): δ 7.35 (d, 2H, H-2 and H-6), 4.01 − 3.97 (m, 2H, NC*H*_2_CH_2_OH), 3.89 (s, 6H, 2x(OC*H*_3_)), 3.49 − 3.44 (m, 2H, NCH_2_C*H*_2_OH), 3.19 (s, 9H, N(C*H*_3_)_3_)). ^13^C NMR (100 MHz, MeOD) δ 175.1 (*C*OO), 148.4 (*C*OH-4), 139.2 (2× (*C*OCH_3_)), 129.6 (*C*COO-1), 108.1 (C-6), 69.05 (N*C*H_2_CH_2_OH), 57.04 (NCH_2_*C*H_2_OH), 56.69 (2× (O*C*H_3_)), 54.70, 54.67, 54.63 (N(*C*H_3_)_3_). ^1^H NMR and ^13^C NMR data agree with those reported in the literature [19].

##### Trimethyl-β-hydroxyethyl-ammonium Vanillate ([Chol]Van)

The preparation of [Chol]Van (99% yield, light yellow solid) was performed according to the general procedure for imidazolium-based ILs. ^1^H NMR (400 MHz, MeOD): δ 7.61 (d, 1H, H-2), 7.50 (dd, 1H, H-6), 6.79 (d, 1H, H-5), 4.01 − 3.96 (m, 2H, NCH_2_C*H*_2_OH), 3.90 (s, 3H, OC*H*_3_), 3.48 − 3.44 (m, 2H, NC*H*_2_CH_2_OH), 3.19 (s, 9H, N(*CH*_3_)_3_). ^13^C NMR (100 MHz, MeOD) δ 175.3 (*C*OO), 150.28 (*C*OH-4), 148.1 (*C*OCH_3_-3), 130.7 (*C*COOH), 124.2 (C-6), 115.30 (C-5), 114.1 (C-2), 69.04 (N*C*H_2_CH_2_OH), 57.02 (NCH_2_*C*H_2_OH), 56.32 (O*C*H_3_), 54.69, 54.66, 54.62 (N(*C*H_3_)_3_). ^1^H NMR and ^13^C NMR data agree with those reported in the literature [12].

#### 3.2.3. General Procedure for the Synthesis of Protic Lignin-Based ILs

**Figure 9:**
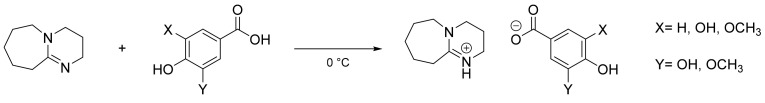
Synthetic steps for [DBU]Gal; [DBU]Syr; [DBU]Van.

An equimolar amount of phenolic acid was added to 1,5-diazabicyclo [5.4.0] undec-7-ene [DBU] at 0 °C without the addition of solvent. The reaction was mixed at 0 °C for 5 min and then the mixture was returned to room temperature. The structure of the compounds was confirmed by ^1^H and ^13^C-NMR and the spectra are reported in the Supplementary Materials. The synthetic steps of the synthesis are reported in Figure 9.

##### 1,5-Diazabicyclo [5.4.0]undec-7-eneium Gallate ([DBU]Gal)

The preparation of [DBU]Gal (99% yield, light yellow solid) was performed according to the general procedure for imidazolium-based ILs. ^1^H NMR (400 MHz, MeOD): δ 7.03 (s, 2H, H-2 e H-6), 3.58 − 3.53 (m, 2H, H-6 DBU), 3.50 (t, 2H, H-4 DBU), 3.32 (t, 2H, H-2 DBU) 2.66–2.61 (m, 2H, H-10 DBU), 2.00 (sept, 2H, H-2 DBU) 1.81 − 1.61 (m, 6H, H-7,8,9 DBU). ^13^C NMR (100 MHz, MeOD) δ 175.4 (*C*OO), 167.5 (N-*C=*N), 145.9 (C-3 e C-5), 137.8 (C-4), 129.6 (C-1), 110.1 (C-2 e C-6), 55.37 (C-6 DBU), 49.54 (C-4 DBU), 39.34 (C-2 DBU), 33.70 (C-10 DBU), 29.91 (C-7 DBU), 27.45 (C-8 DBU), 24.91 (C-9 DBU), 20.41 (C-3 DBU).

##### 1,5-Diazabicyclo [5.4.0]undec-7-eneium Syringate ([DBU]Syr)

The preparation of [DBU]Syr (99% yield, light yellow solid) was performed according to the general procedure for imidazolium-based ILs. ^1^H NMR (400 MHz, MeOD): δ 7.35 (s, 2H, H-2 e H-6), 3.89 (s, 3H, OC*H*_3_), 3.61 − 3.54 (m, 2H, H-6 DBU), 3.52 (t, 2H, H-4 DBU), 3.34 (t, 2H, H-2 DBU) 2.70 − 2.613 (m, 2H, H-10 DBU), 2.02 (sept, 2H, H-2 DBU) 1.83 − 1.64 (m, 6H, H-7,8,9 DBU). ^13^C NMR (100 MHz, MeOD) δ 175.1 (*C*OO), 167.5 (N-*C=*N), 148.4 (C-3 e C-5), 139.1 (C-4), 129.7 (C-1), 108.1 (C-2 e C-6), 56.71 (2×(O*C*H_3_)), 55.35 (C-6 DBU), 49.55 (C-4 DBU), 39.34 (C-2 DBU), 33.70 (C-10 DBU), 29.92 (C-7 DBU), 27.46 (C-8 DBU), 24.96 (C-9 DBU), 20.41 (C-3 DBU).

##### 1,5-Diazabicyclo [5.4.0]undec-7-eneium Vanillate ([DBU]Van)

The preparation of [DBU]Van (99% yield, light yellow solid) was performed according to the general procedure for imidazolium-based ILs. 7.60 (d, 1H, H-2), 7.49 (dd, 1H, H-6), 6.77 (d, 1H, H-5), 3.90 (s, 3H, OC*H*_3_), 3.60 − 3.55 (m, 2H, H-6 DBU), 3.51 (t, 2H, H-4 DBU), 3.33 (t, 2H, H-2 DBU) 2.69 − 2.63 (m, 2H, H-10 DBU), 2.01 (sept, 2H, H-2 DBU) 1.80 − 1.61 (m, 6H, H-7,8,9 DBU). ^13^C NMR (100 MHz, MeOD) δ 175.2 (*C*OO), 167.5 (N-*C=*N), 150.0 (*C*OH-4), 148.1 (*C*OCH_3_-3), 130.7 (*C*COOH), 124.2 (C-6), 115.20 (C-5), 114.2 (C-2), 56.35 (2x(O*C*H_3_)), 55.32 (C-6 DBU), 49.53 (C-4 DBU), 39.32 (C-2 DBU), 33.65 (C-10 DBU), 29.91 (C-7 DBU), 27.45 (C-8 DBU), 24.91 (C-9 DBU), 20.40 (C-3 DBU).

### 3.3. Characterization Methods

#### 3.3.1. Thermal Gravimetric Analysis (TGA)

The thermal stability of ILs was investigated by using thermal gravimetric analysis (TA Instruments Q500 TGA, New Castle, DE, USA). The instrument was calibrated using weight standards (1 g and 100 mg, TA Instruments) and the temperature calibration was performed using nickel standard (TA Instruments). The IL samples (10–15 mg) were heated at 40 °C in a platinum crucible for the drying procedure and maintained in N_2_ flux (90 mL/min) for 30 min. Then, the samples were heated from 40 to 600 °C with a heating rate of 10 °C/min under nitrogen (90 mL/min) and maintained at 600 °C for 3 min. Mass change was recorded as a function of temperature and time. TGA experiments were carried out in duplicate.

#### 3.3.2. Differential Scanning Calorimetry (DSC)

The thermal behavior of LevILs was analyzed using a differential scanning calorimeter (TA DSC, Q250, USA, temperature accuracy ±0.05 °C, temperature precision ±0.008 °C, enthalpy precision ± 0.08%). Dry high-purity N_2_ gas was flown through the sample at a flow rate of 50 mL/min. A weight of approximately 3 mg of each sample was placed in a hermetically sealed aluminum crucible with a pinhole and the phase behavior was studied under a nitrogen atmosphere in the temperature range from 90 to 120 °C at a heating rate of 10 °C/min. Temperature calibration was performed considering the heating rate dependence of the onset melting peak temperature of indium. The enthalpy was also calibrated using indium (melting enthalpy Δ_m_*H* = 28.71 J g^−1^). DSC experiments were performed in duplicate. *T*_g_ was obtained by taking the midpoint of the heat capacity change on heating from a glass to a liquid. *T*_m_ was taken as the peak temperature of the endothermic peak on heating. Peak temperatures were chosen instead of onset temperatures because of the complexity of the thermograms.

#### 3.3.3. PEIS and CPP

BioLogicVMP3 multichannel potentiostat and EC-Lab^®^ V11.42 software were used for PEIS and CPP experiments. The setup for the test was the following: a three-electrode cell where the counter electrode (CE) was graphite, and the working electrode (WE) was a rod of mild steel AS1020 with a surface of 1.1 cm^2^ and equipped with an Ag/AgCl reference electrode. The working electrode was polished using polishing paper with three different grits: 240, 800, and 1200 to assure that all the samples have a comparable surface.

The inhibitor efficiency was extrapolated through Tafel plot according to Equation (1):

(1) $$\eta =\frac{i_{corr\;control-i_{corr\left(i\right)}}}{i_{corr\;control}\;}$$

where *i_corr control_* is the current density measured when the sample is immersed in the control solution (0.01 NaCl) and *i_corr_*_(*i*)_ is the current density measured when the sample is immersed in inhibited solution.

Since the curves can be considered linear in the range of 10–25 mV for either side, the extrapolations were made over this range for every sample, and they were calculated using the software Origin© 2022b 9.9.

#### 3.3.4. Scanning Electron Microscopy (SEM) and Energy-Dispersive X-ray Spectroscopy (EDS)

A JSM-IT300 LV SEM instrument equipped with an Oxford X-Max 50 mm^2^ EDS detector at 15 kV was used to obtain micrographs of the surfaces after immersion. The EDS detector was used with an accelerating voltage of 40 kV and the corresponding spectra were elaborated using the software AZtec 6.0 by Oxford Instruments, Abingdon, UK.

#### 3.3.5. Optical Microscope

A Leica MZ 7 optical microscope was used to observe surfaces of the samples after 24 h of immersion, the images were elaborated through the software LAS V4.0.

#### 3.3.6. Optical Profilometer

Three-dimension surface profilometry was performed on a Bruker Contour GT-K1 3D optical microscope on samples immersed for 24 h in the respective solution that had the corrosion products removed. The corrosion products were removed following ASTM G1-03 (2011) [20], chemical cleaning procedure Table 1.1 designation C.3.5. Every scan was elaborated using the software Vision 64, and the average roughness S_a_ (arithmetic average of the 3D roughness) was extrapolated for each of the 630 × 470 μm scans. To have the most representative values, measurements were taken in 3 different parts of the sample that did not contain evident defects.

## 4. Conclusions

In this article, the effect of cations and anions in novel ionic liquids as corrosion inhibitors was investigated. All the new compounds were obtained as solids (except [Emim]Van) with melting temperature below 200 °C, with the class of DBU that degrades before melting. From the thermal characterizations, it emerged that they present different behaviors, especially in the case of gallate anions, which showed two distinct degradation events, with a third one appearing when combined with choline cations. The choline cation seems to cause the presence of an additional degradation event also in combination with the syringate anion.

From preliminary electrochemical tests, the DBU cation and vanillate anion showed low anticorrosion properties and, for this reason, they were discarded.

The gallate anion was less effective as part of the IL inhibitors, most likely as it takes part in the formation of a soluble complex with iron, which does not show any protection against corrosion, as was confirmed by surface analysis.

The syringate anion in solution showed an increase in impedance when combined with each cation and a significant decrease in the corrosion current. From the SEM and EDS, it was possible to see both the ILs [Chol]Syr and [Emim]Syr deposits containing carbon and nitrogen atoms, which confirmed the presence of the inhibitor on the surface. This result demonstrates how the inhibitor was able to interact with the surface of the steel and shut down the corrosion process.

Even if further studies are needed to confirm and clarify the degradation mechanism of these ILs and their anticorrosion behaviors, it can be stated that the fully biobased [Chol]Syr was the most effective corrosion inhibitor, presenting a reduction in corrosion current from 1.66 to 0.066 µA/cm^2^ when the IL was added to a 0.01 M NaCl solution.

## Figures and Tables

**Table 1:** *T*_start(5%)_, *T*_onset_, and *T*_peak_ of the investigated lignin-based ILs under a nitrogen atmosphere and at 10 °C/min heating rate.

	TGA			DSC	
ILs	*T*_start_ (°C)	*T*_onset_ (°C)	*T*_peak_ (°C)	*T*_m_ (°C)	*T*_g_ (°C)
[Emim]Gal	201.0	189.8 253.5	202.4 280.33	-	-
[Emim]Syr	175.8	204.2	225.21	148.0	5.99
[Emim]Van	174.0	204.1	230.69		−1.17
[Chol]Gal	201.5	215.4	263.0	135.8	22.29
[Chol]Syr	178.2	186.5	206.4	157.4	18.63
[Chol]Van	204.7	212.14	223.4	181.6	-
[DBU]Gal	201.0	201.03 264.91	214.2 284.3	76.37 ^a^	-
[DBU]Syr	190.0	202.3	219.7	-	-
[DBU]Van	204.1	210.9	221.4	-	-

^a^ solid–solid transition.

**Table 2:** Corrosion parameters obtained from CPP using Tafel extrapolation, in control and inhibited solutions after 24 h of immersion.

Solution	MW	Concentration (mM)	E_corr_ (mV)	*i*_corr_ (µA/cm^2^)	IE (%)
**control**		100	−604	1.457	-
**[Emim]Gal**		3	−695	1.032	29
	280.28	5	−689	0.923	37
		10	−712	0.830	43
**[Emim]Syr**		3	−471	1.170	20
	308.33	5	−468	0.974	33
		10	−522	0.312	79
**[Chol]Gal**		3	−696	1.051	28
	273.29	5	−684	0.733	49
		10	−706	0.902	38
**[Chol]Syr**		3	−486	1.151	21
	301.34	5	−578	0.227	85
		10	−491	0.066	96

## Data Availability

Data sharing not applicable to this article as no datasets were generated or analyzed during the current study.

## Supplementary Materials

The following supporting information can be downloaded at: https://www.mdpi.com/article/10.3390/molecules28145568/s1, Figure S1 Bode plot (left) and Cyclic Potentiodynamic Polarization (right) results of AS1020 mild steel after 24 h at OCV in control (0.01 M NaCl; dotted line) and inhibited solutions (0.01 M NaCl + 5 mM inçhibitor); Figure S2 EDS data of pristine mild steel; Figure S3 EDS data of mild steel immersed in 0.01 M NaCl + [Emim]Gal 10 mM (a); 0.01 M NaCl + [Emim]Syr 10 mM (b) after 24 h; Figure S4 EDS data of 3 different zones (1,4: mild steel surface; 2,3: inhibitor deposits) of mild steel immersed in 0.01 M NaCl + [Chol]Gal 10 mM (b); 0.01 M NaCl + [Chol]Syr 10 mM (b) after 24 h; Table S1. Average area roughness of mild steel samples immersed in the inhibited solutions containing 0.01 M NaCl and the inhibitor 10 mM, compared to pristine control immersed in 0.01 M NaCl; Figure S5 ^1^H NMR and ^13^C NMR spectra of [Emim]Gal; Figure S6 ^1^H NMR and ^13^C NMR spectra of [Emim]Syr; Figure S7 ^1^H NMR and ^13^C NMR spectra of [Emim]Van; Figure S8 ^1^H NMR and ^13^C NMR spectra of [Chol]Gal; Figure S9 ^1^H NMR and ^13^C NMR spectra of [Chol]Syr; Figure S10 ^1^H NMR and ^13^C NMR spectra of [Chol]Van; Figure S11 ^1^H NMR and ^13^C NMR spectra of [DBU]Gal; Figure S12 ^1^H NMR and ^13^C NMR spectra of [DBU]Syr; Figure S13 ^1^H NMR and ^13^C NMR spectra of [DBU]Van; Figure S14 Thermogravimetric and derivative curves of [Emim]Gal; Figure S15 Thermogravimetric and derivative curves of [Emim]Syr; Figure S16 Thermogravimetric and derivative curves of [Emim]Van; Figure S17 Thermogravimetric and derivative curves of [Chol]Gal; Figure S18 Thermogravimetric and derivative curves of [Chol]Syr; Figure S19 Thermogravimetric and derivative curves of [Chol]Van; Figure S20 Thermogravimetric and derivative curves of [DBU]Gal; Figure S21 Thermogravimetric and derivative curves of [DBU]Syr; Figure S22 Thermogravimetric and derivative curves of [DBU]Van; Figure S23 DSC curve of [Emim]Gal; Figure S24 DSC curves of 1st and 2nd heating run of [Emim]Syr; Figure S25 DSC curve of [Emim]Van; Figure S26 DSC curves of 1st and 2nd heating run of [Chol]Gal; Figure S27 DSC curves of 1st and 2nd heating run of [Chol]Syr; Figure S28 DSC curve of [Chol]Van; Figure S29 DSC curves of [DBU]Gal; Figure S30 DSC curves of [DBU]Syr; Figure S31 DSC curves of [DBU]Van.
