# Supplementary material for: Lignin-Derivative Ionic Liquids as Corrosion Inhibitors
Source: Molecules. 2023 Jul 21;28(14):5568. doi: 10.3390/molecules28145568 (PMC10384825; doi:10.3390/molecules28145568)
Supplement: Supplementary file 1 [file molecules-28-05568-s001.zip › molecules-2501473-supplementary.pdf]

molecules

Supplementary

Lignin-Derivative Ionic Liquids as Corrosion Inhibitors

Sharon Monaci [1,2], Daniela Minudri [1], Lorenzo Guazzelli [3], Andrea Mezzetta [3], David Mecerreyes [1,4], Maria Forsyth [1,2,4] and Anthony Somers [2,*]

[1] POLYMAT, University of the Basque Country UPV/EHU, 20018 Donostia-San Sebastian, Spain; smonaci@deakin.edu.au (S.M.); daniela.minudri@polymat.eu (D.M.); david.mecerreyes@ehu.es (D.M.); maria.forsyth@deakin.edu.au (M.F.)

[2] Institute for Frontier Materials, Deakin University, Burwood, VIC 3125, Australia

[3] Dipartimento di Farmacia, Università di Pisa, Via Bonanno Pisano 33, 56126 Pisa, Italy; lorenzo.guazzelli@unipi.it (L.G.); andrea.mezzetta@unipi.it (A.M.)

[4] Ikerbasque, Basque Foundation for Science, 48009 Bilbao, Spain

* Correspondence: anthony.somers@deakin.edu.au

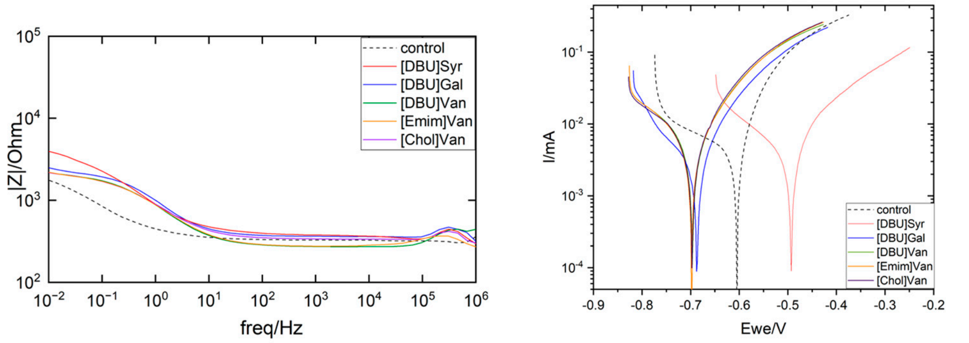

Figure S1 Bode plot (left) and Cyclic Potentiodynamic Polarization (right) results of AS1020 mild steel after 24h at OCV in control (0.01M NaCl; dotted line) and inhibited solutions (0.01M NaCl + 5 mM inhibitor).

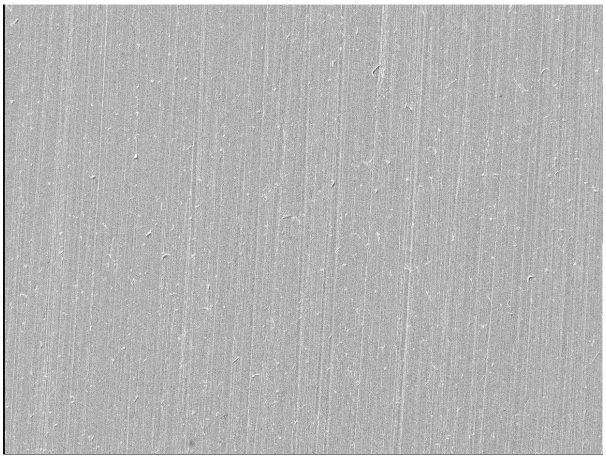

	(Wt%)
Fe	95.0
**O**	0.0
**C**	5.0
**N**	-

Figure S2 EDS data of pristine mild steel.

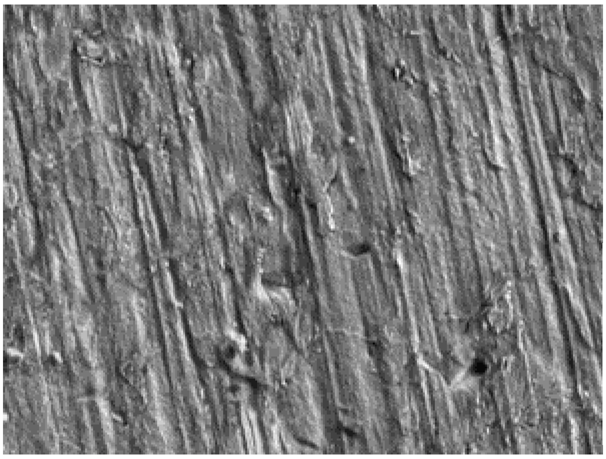

	(Wt%)
Fe	91.5
**O**	7.0
**C**	1.5
**N**	-

(a)

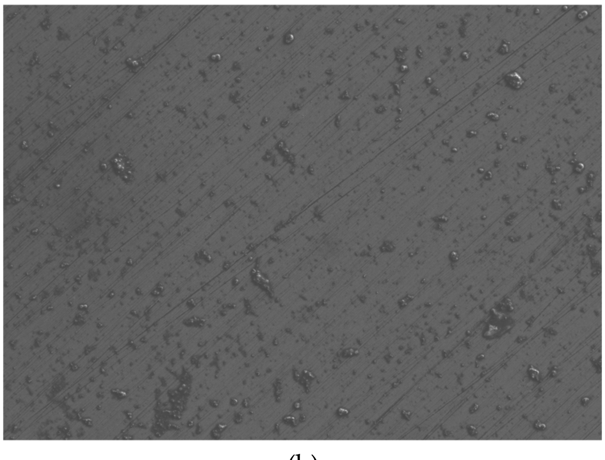

	(Wt%)
Fe	67.6
**O**	1.8
**C**	30.0
**N**	-

(b)

Figure S3 EDS data of mild steel immersed in 0.01M NaCl + [Emim]Gal 10 mM (a); 0.01M NaCl + [Emim]Syr 10 mM (b) after 24h.

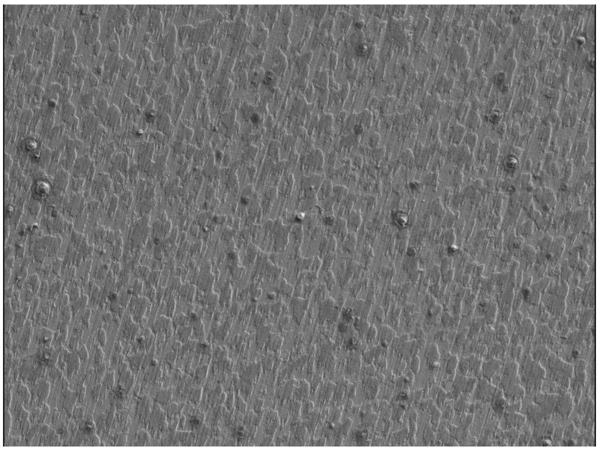

	(Wt%)
Fe	91.4
**O**	3.1
**C**	5.0
**N**	0.5

(a)

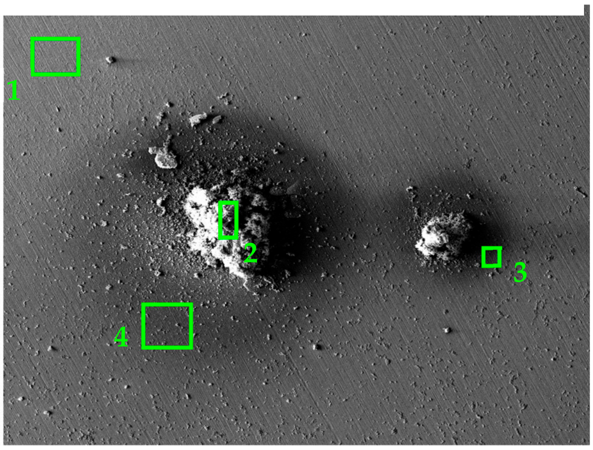

	1	*2*	*3*	*4*
		(Wt%)		
Fe	91.8	34.2	25.0	83.1
**O**	1.0	24.3	25.6	8.3
**C**	6.5	36.0	45.1	8.1
**N**	0.7	5.3	4.2	0.4

(b)

Figure S4 EDS data of 3 different zones (1,4: mild steel surface; 2,3: inhibitor deposits) of mild steel immersed in 0.01M NaCl + [Chol]Gal 10 mM (b); 0.01M NaCl + [Chol]Syr 10 mM (b) after 24h.

Table S1. Average area roughness of mild steel samples immersed in the inhibited solutions containing 0.01M NaCl and the inhibitor 10 mM, compared to pristine control immersed in 0.01M NaCl.

	**Sa (μm)**	**σ**
**Pristine control**	0.101	0.005
**[Emim]Gal**	0.27	0.04
**[Emim]Syr**	0.24	0.03
**[Chol]Gal**	0.161	0.004
**[Chol]Syr**	0.109	0.007

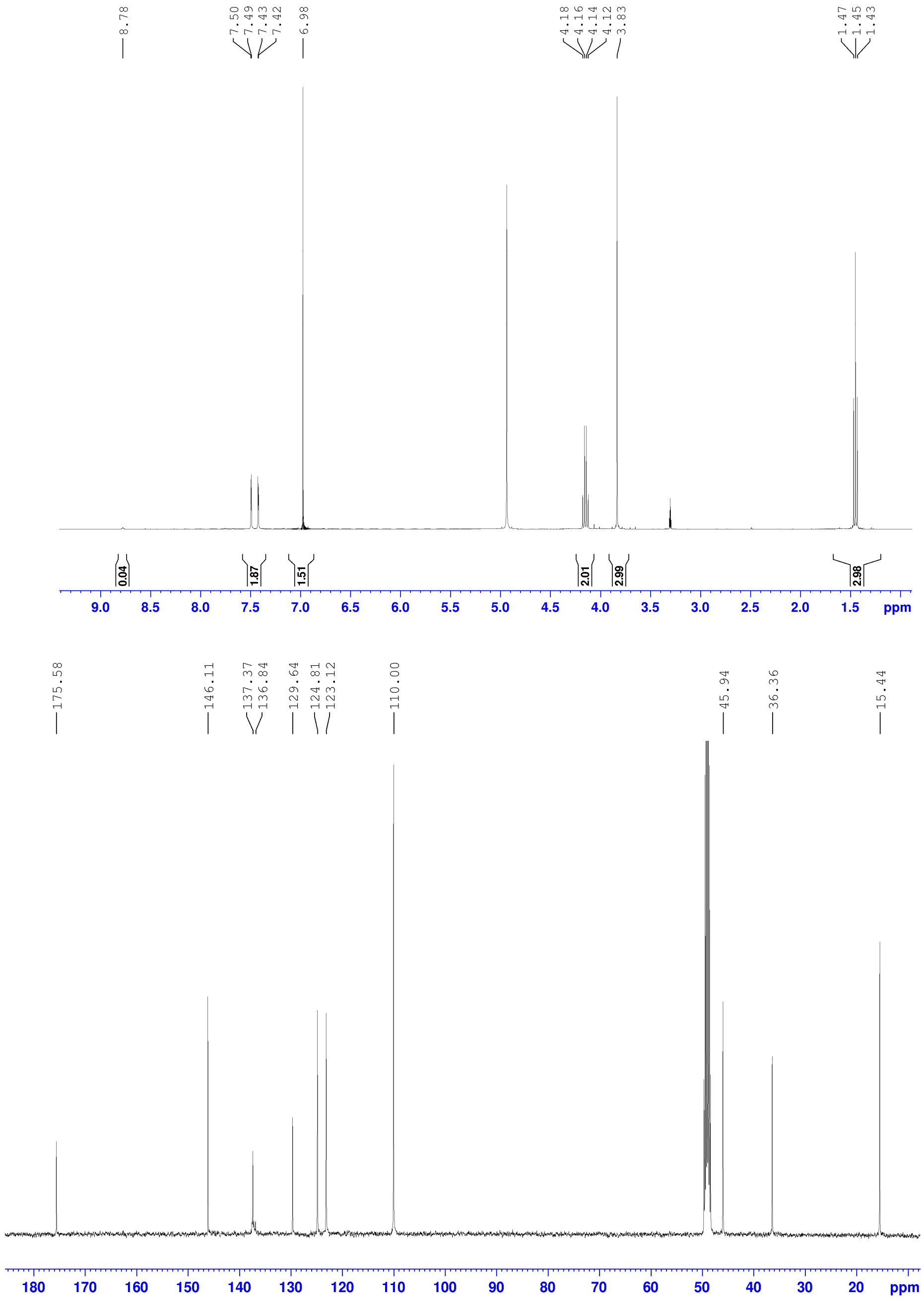

Figure S5 ^{1}H NMR and ^{13}C NMR spectra of [Emim]Gal

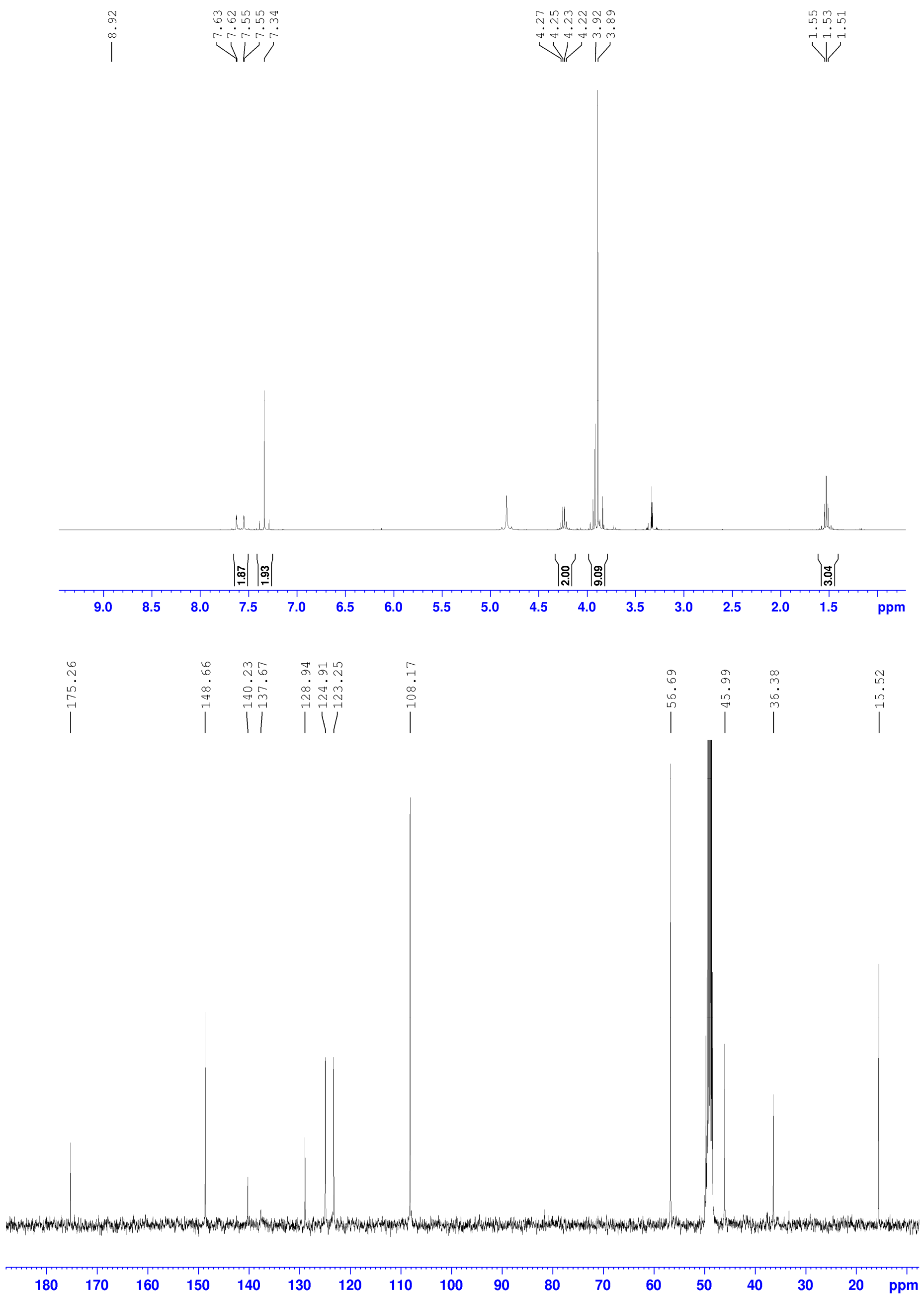

Figure S6 ^{1}H NMR and ^{13}C NMR spectra of [Emim]Syr

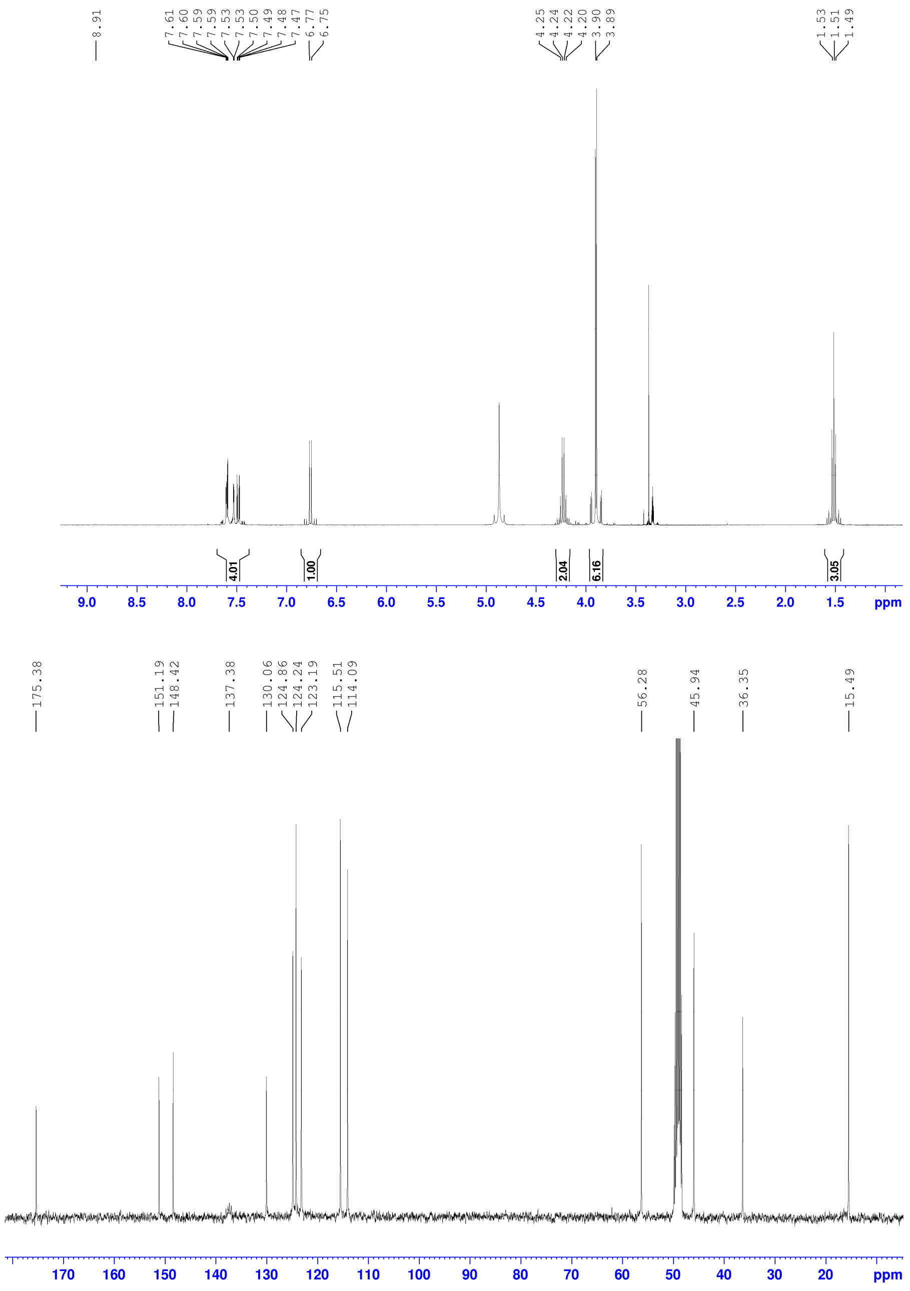

Figure S7 ^{1}H NMR and ^{13}C NMR spectra of [Emim]Van

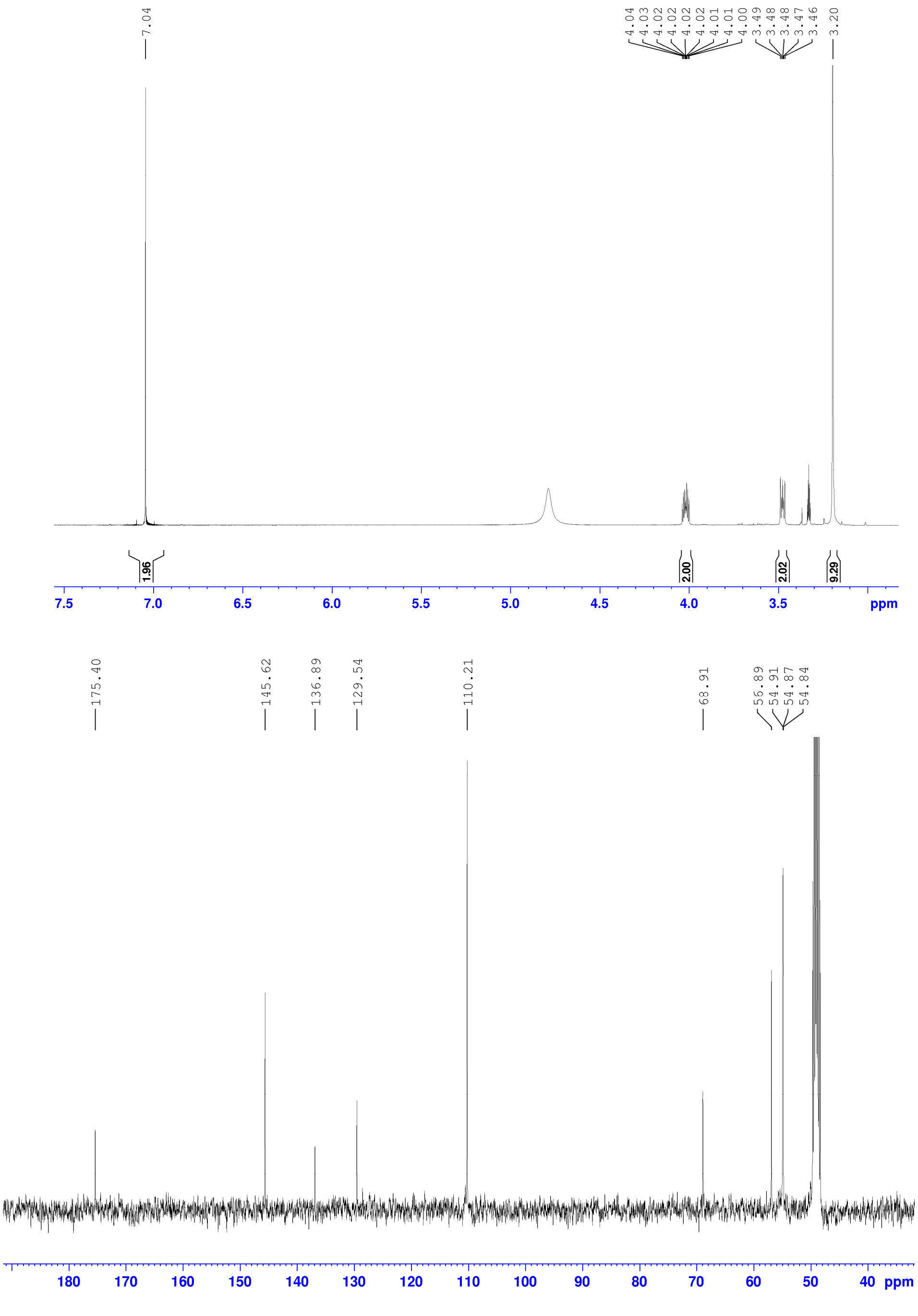

Figure S8 ^{1}H NMR and ^{13}C NMR spectra of [Chol]Gal

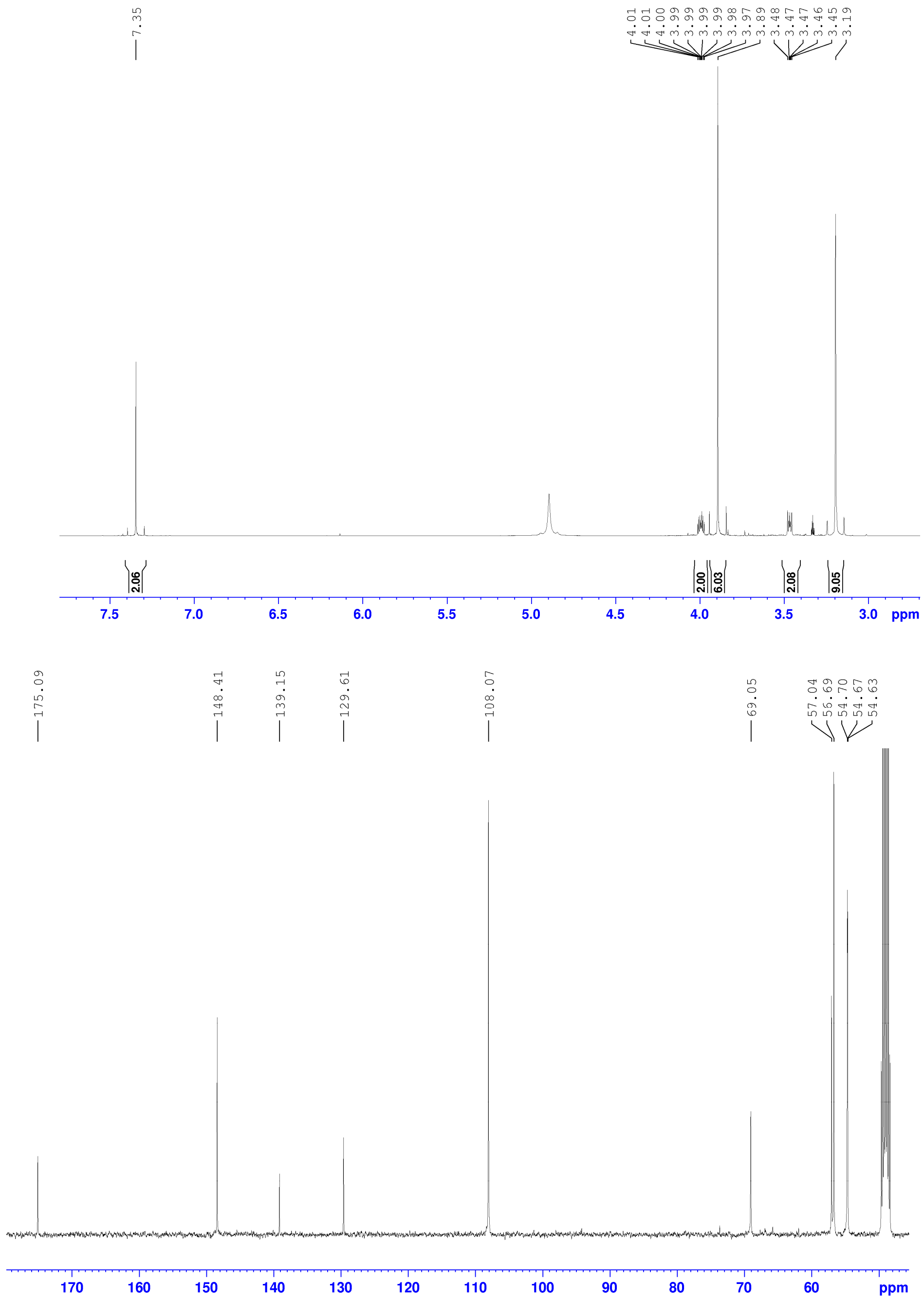

Figure S9 ^{1}H NMR and ^{13}C NMR spectra of [Chol]Syr

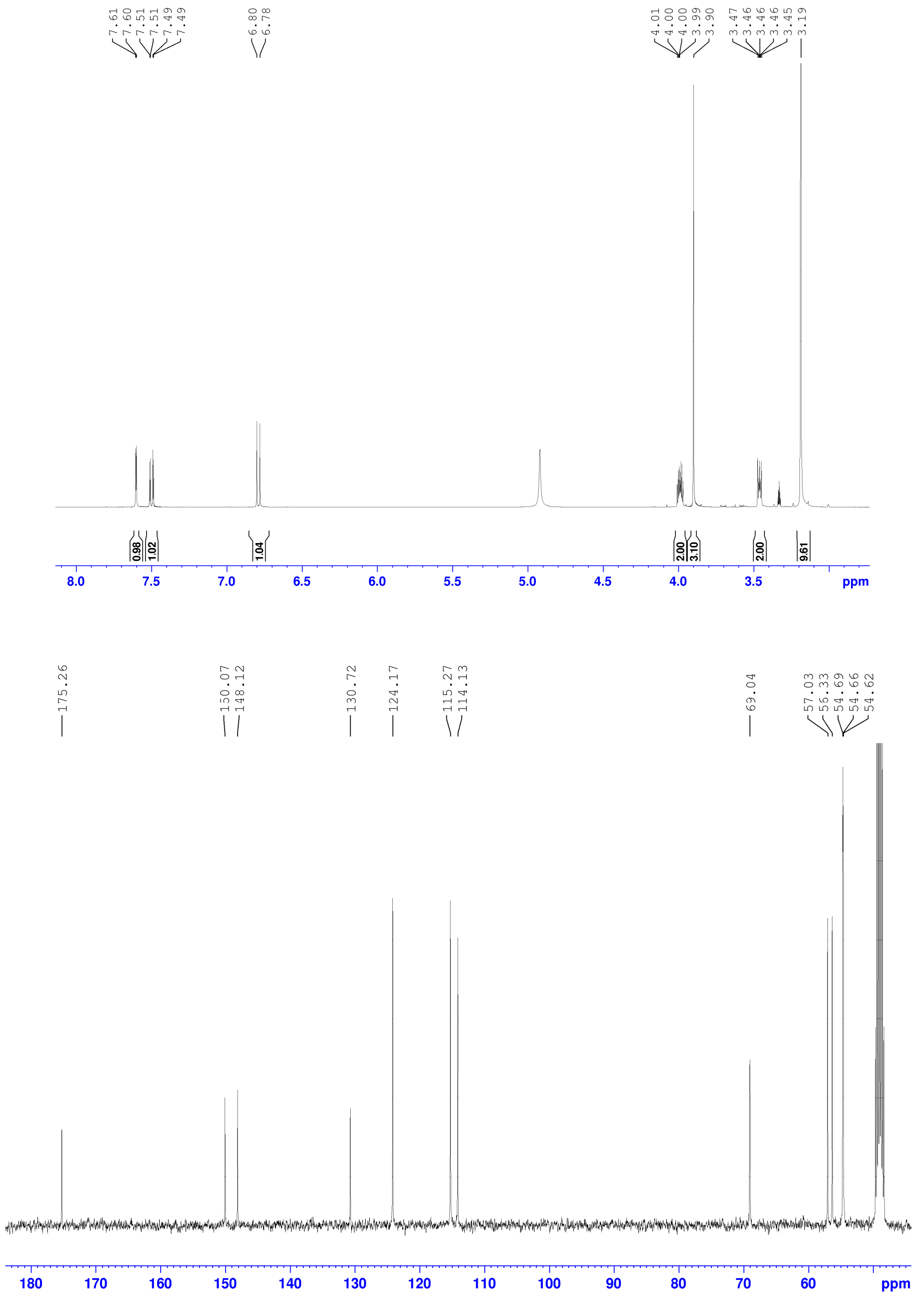

Figure S10 ^{1}H NMR and ^{13}C NMR spectra of [Chol]Van

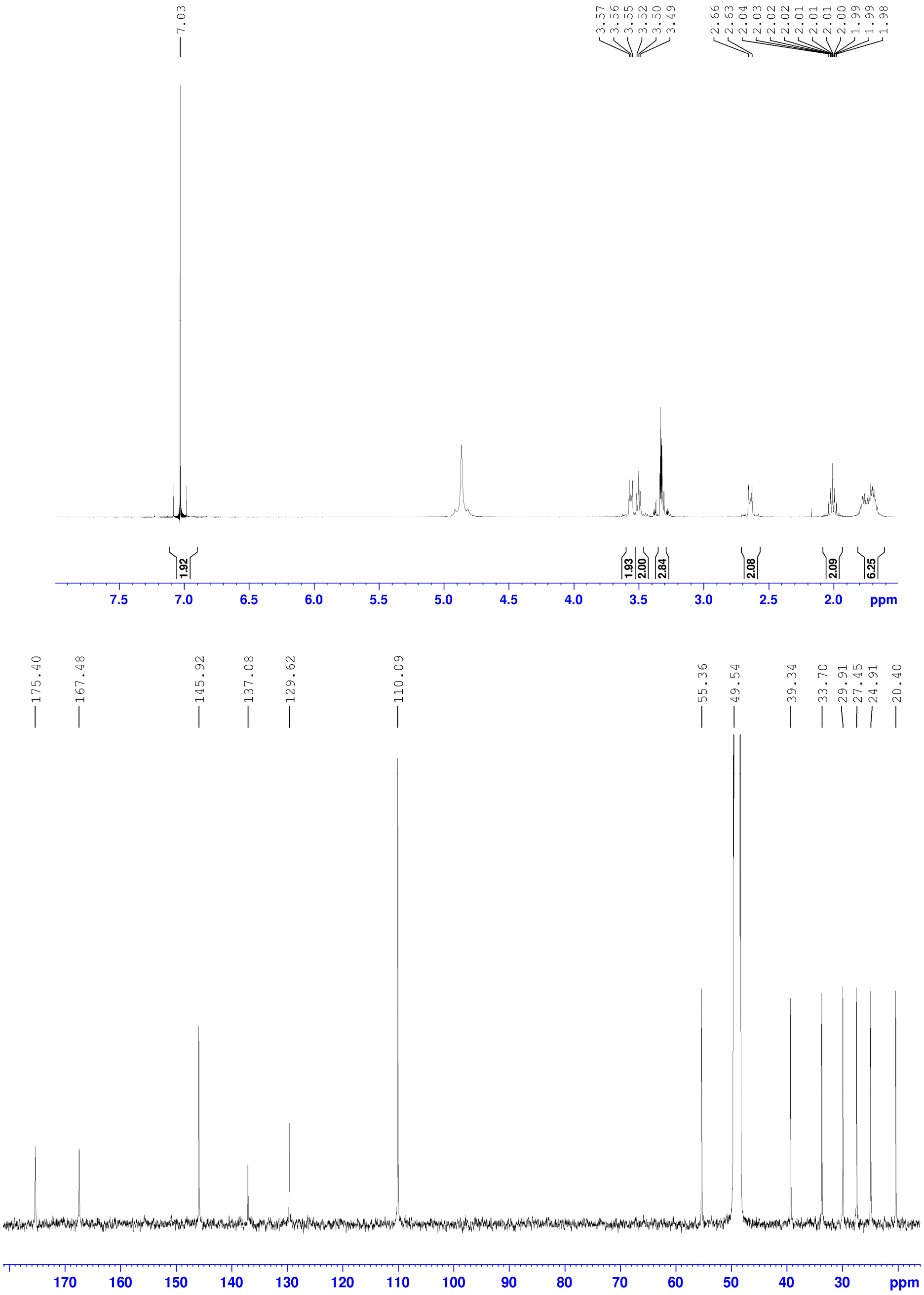

Figure S11 ^{1}H NMR and ^{13}C NMR spectra of [DBU]Gal

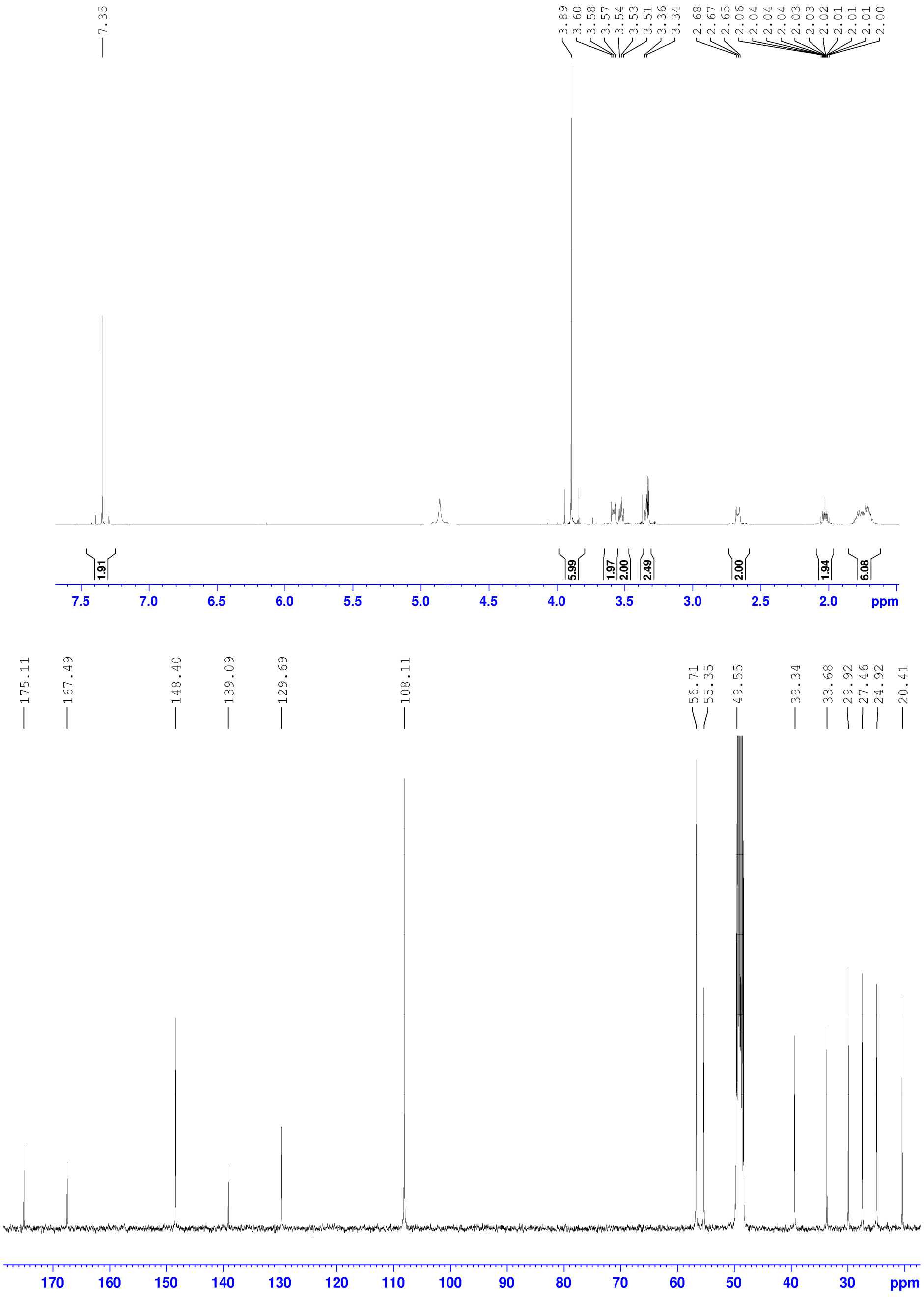

Figure S12 ^{1}H NMR and ^{13}C NMR spectra of [DBU]Syr

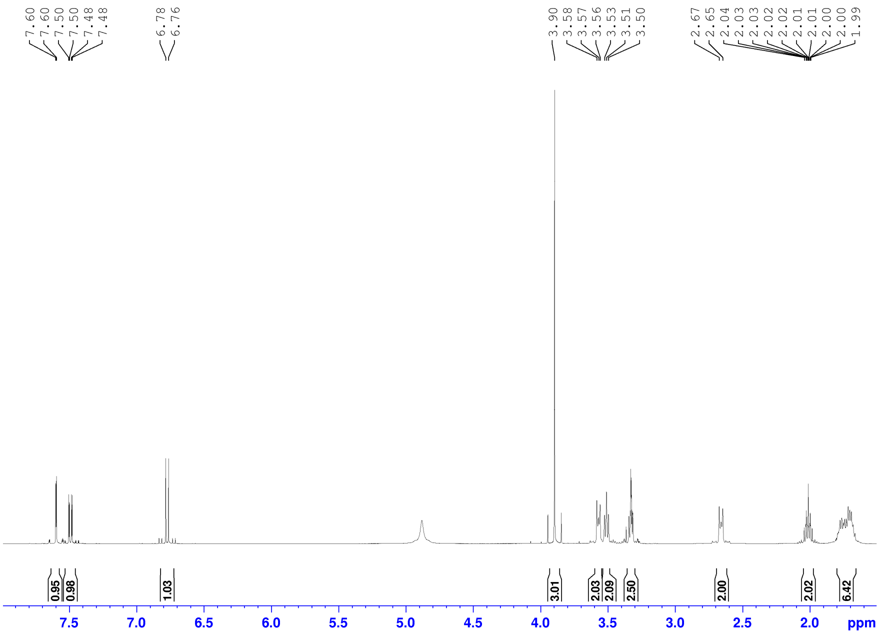

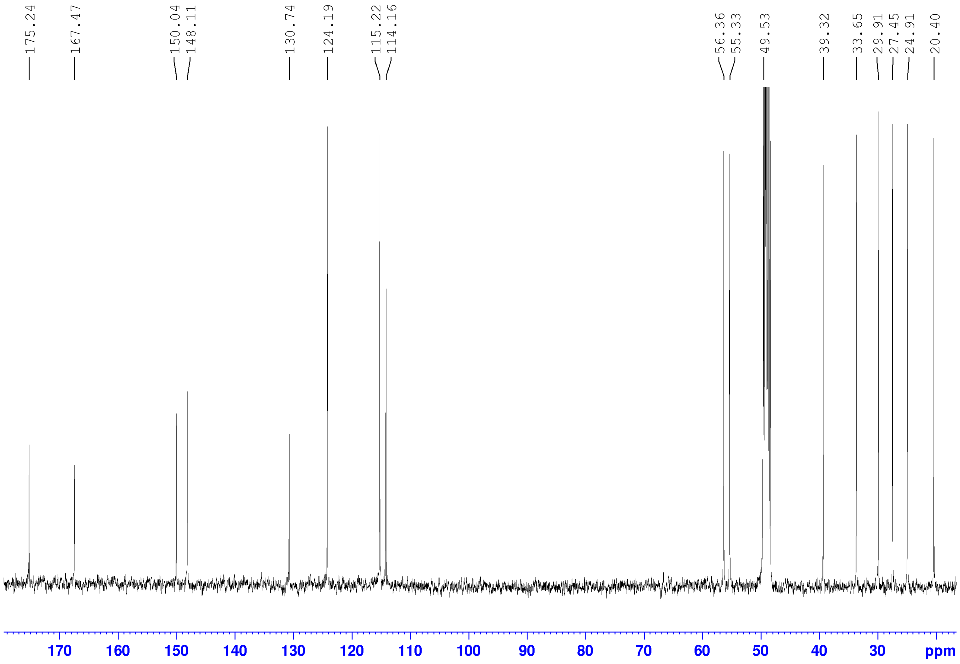

Figure S13 ^{1}H NMR and ^{13}C NMR spectra of [DBU]Van

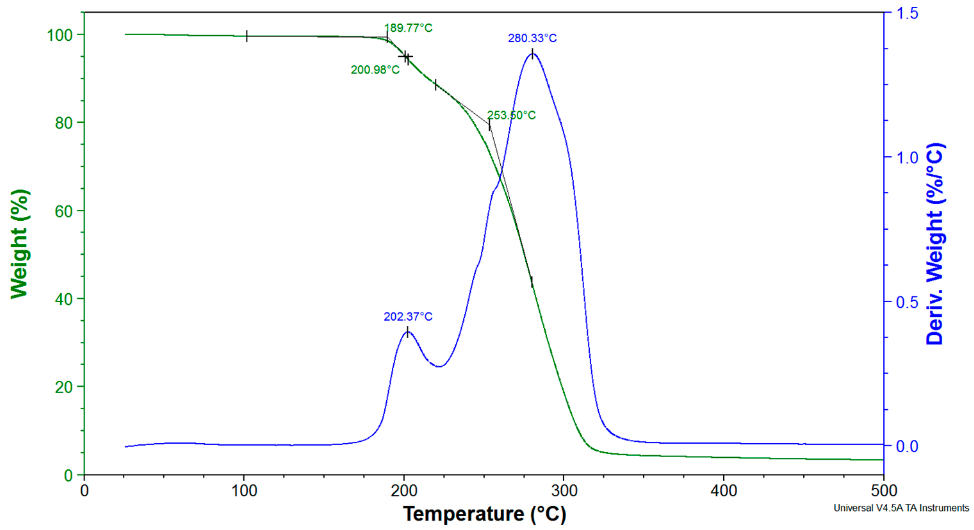

Figure S14 Thermogravimetric and derivative curves of [Emim]Gal

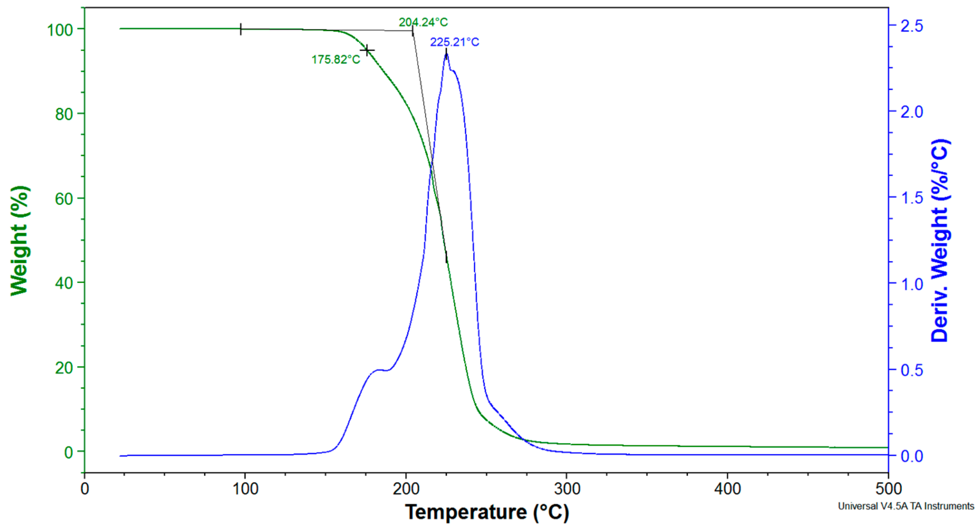

Figure S15 Thermogravimetric and derivative curves of [Emim]Syr

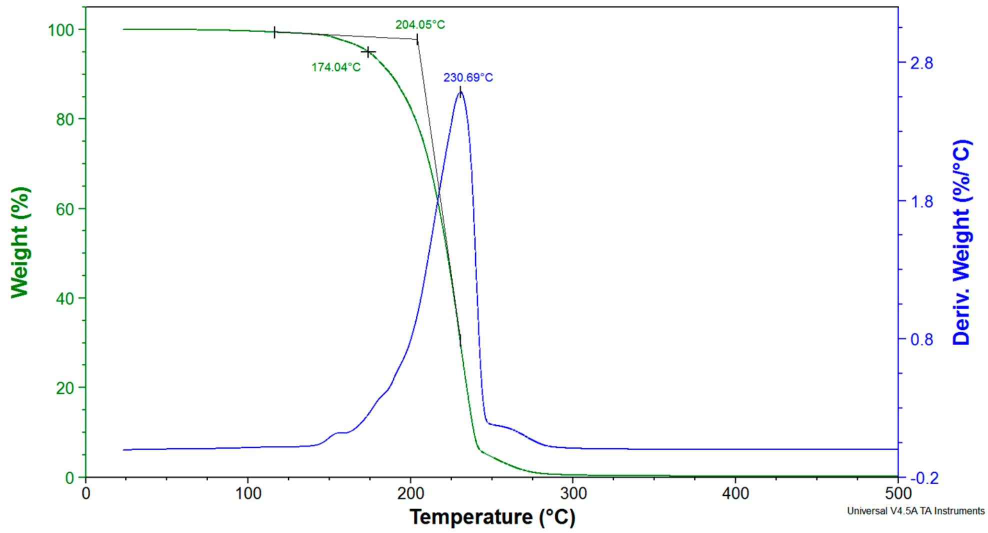

Figure S16 Thermogravimetric and derivative curves of [Emim]Van

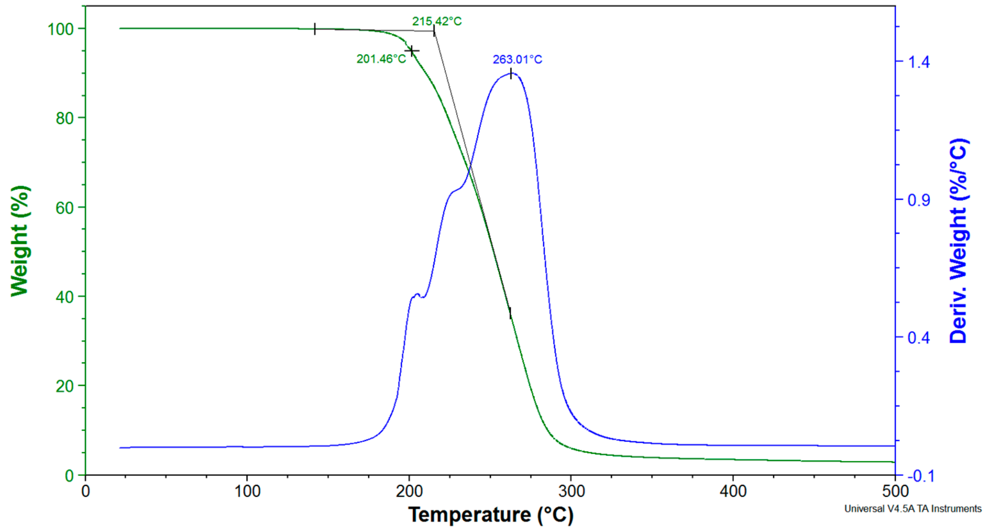

Figure S17 Thermogravimetric and derivative curves of [Chol]Gal

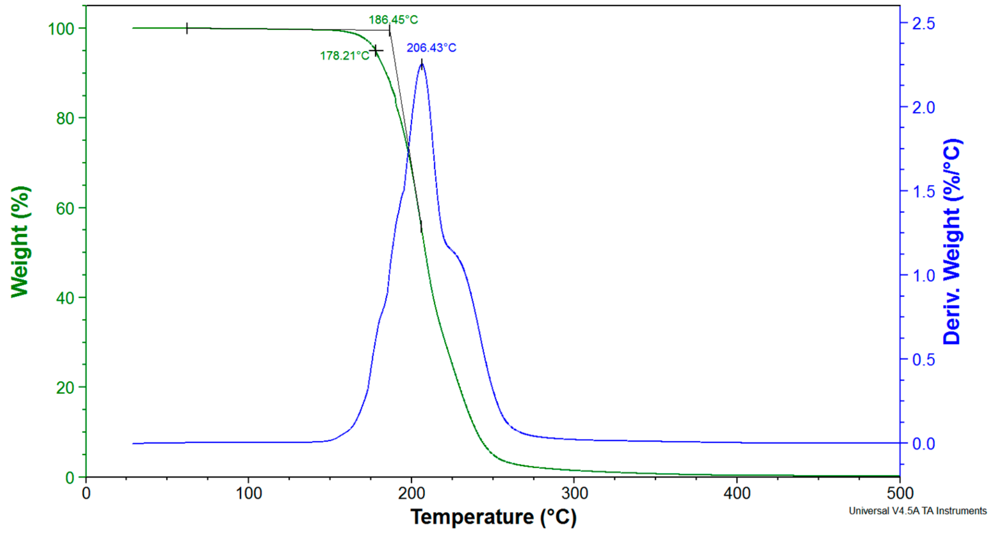

Figure S18 Thermogravimetric and derivative curves of [Chol]Syr

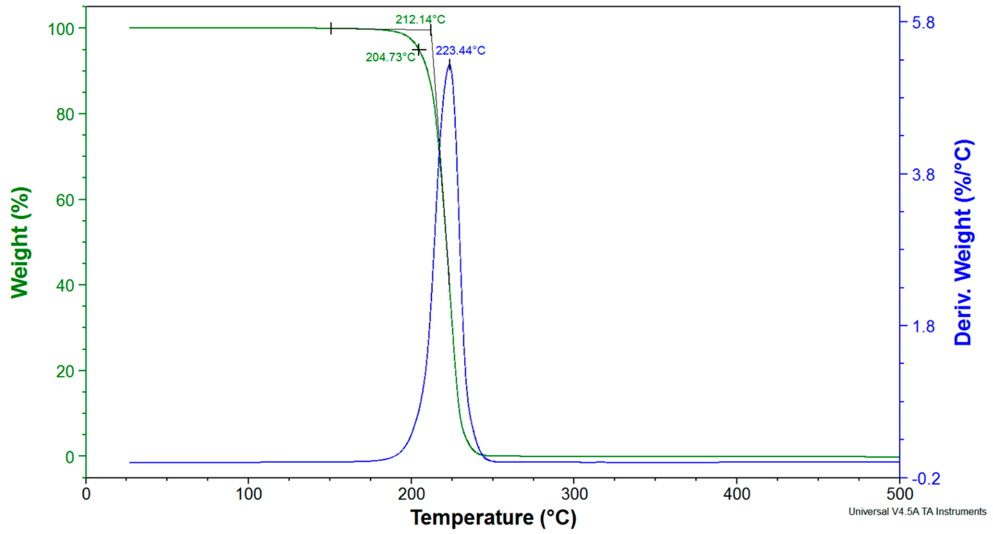

Figure S19 Thermogravimetric and derivative curves of [Chol]Van

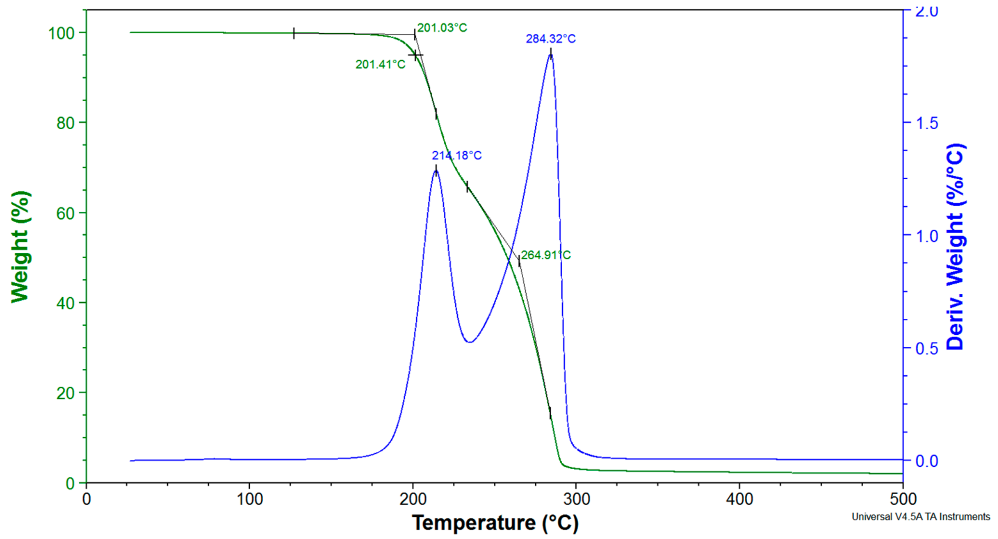

Figure S20 Thermogravimetric and derivative curves of [DBU]Gal

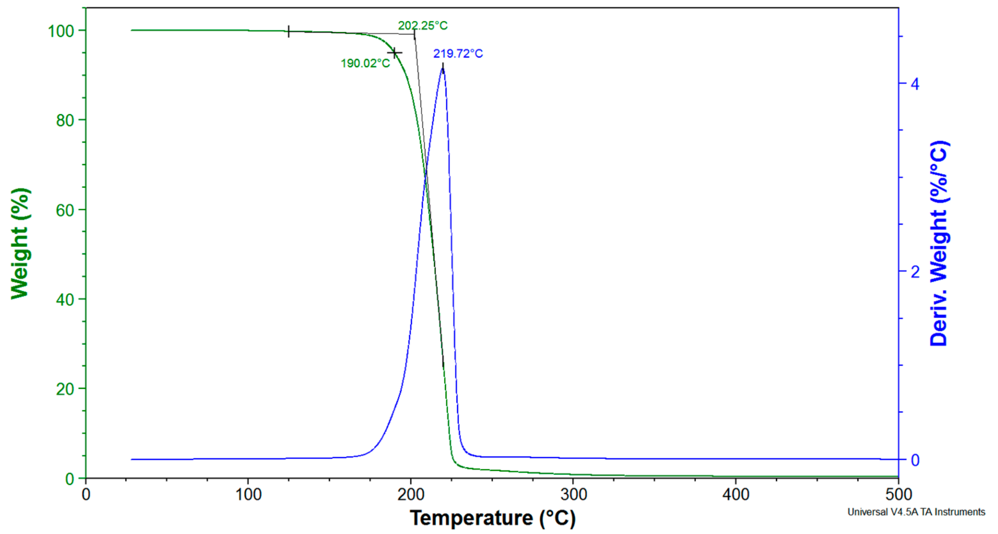

Figure S21 Thermogravimetric and derivative curves of [DBU]Syr

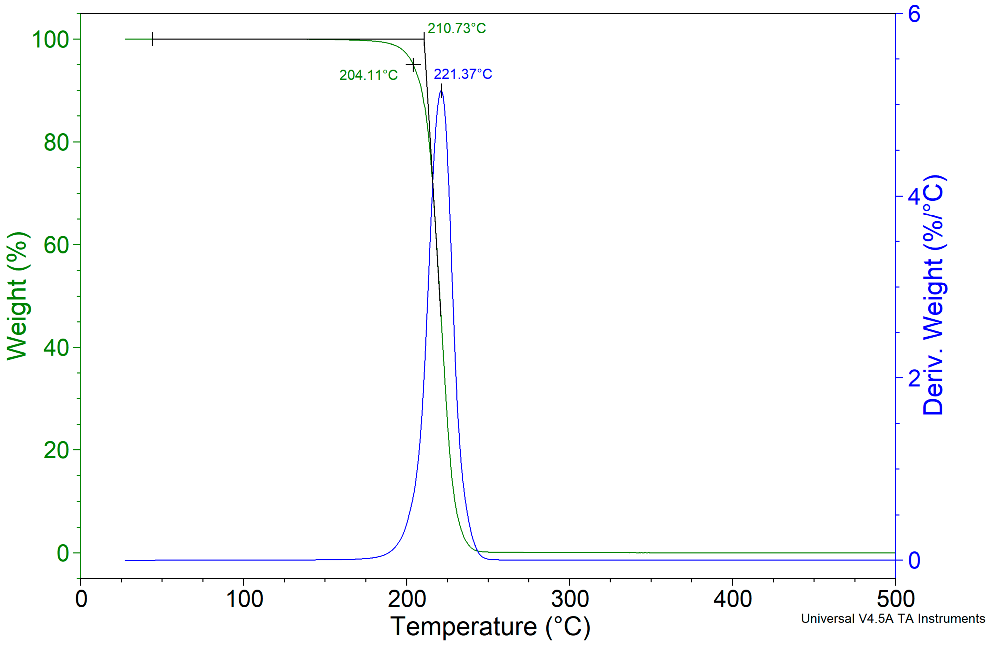

Figure S22 Thermogravimetric and derivative curves of [DBU]Van

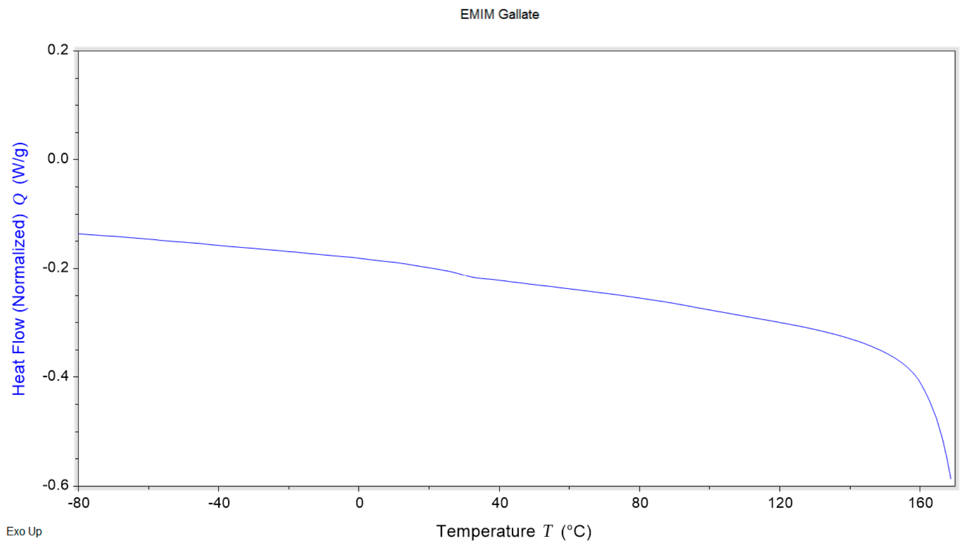

Figure S23 DSC curve of [Emim]Gal

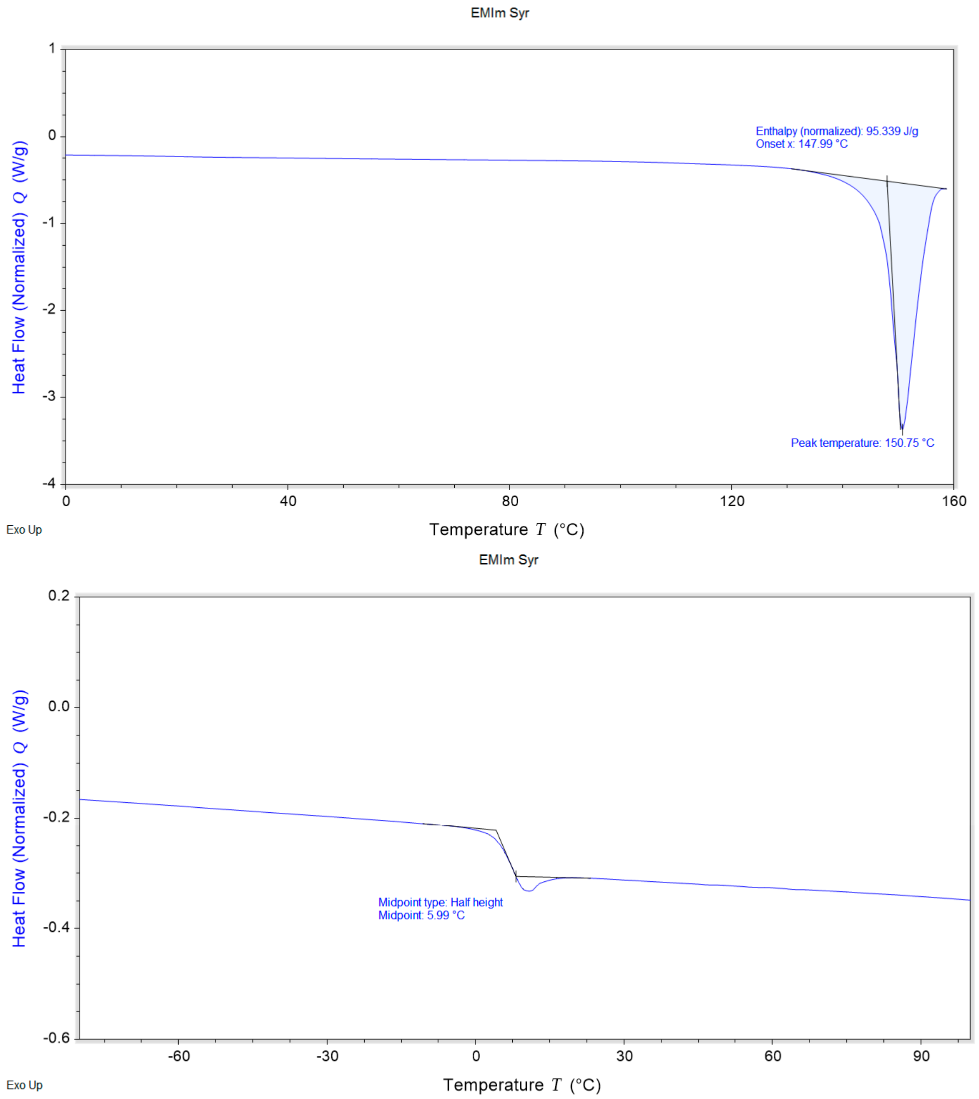

Figure S24 DSC curves of 1st and 2nd heating run of [Emim]Syr

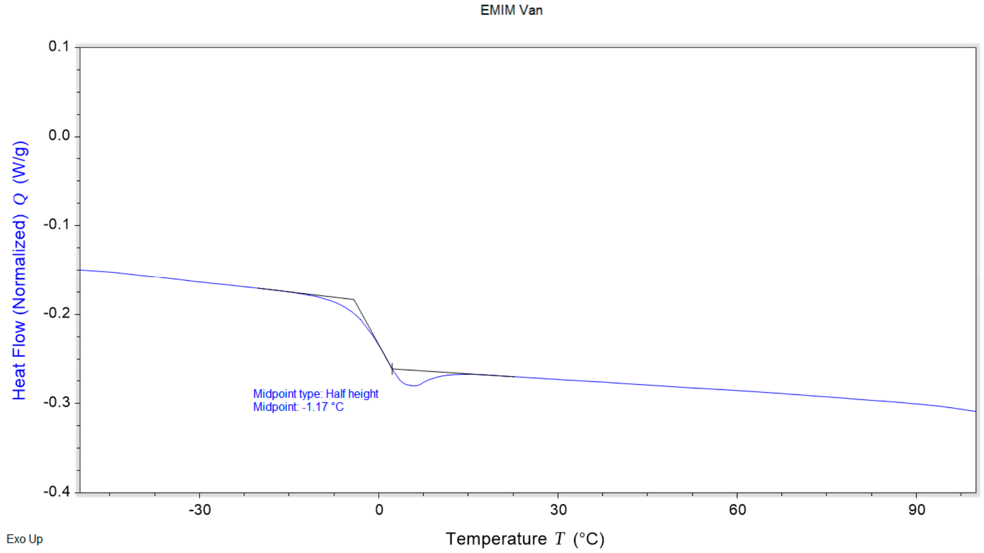

Figure S25 DSC curve of [Emim]Van

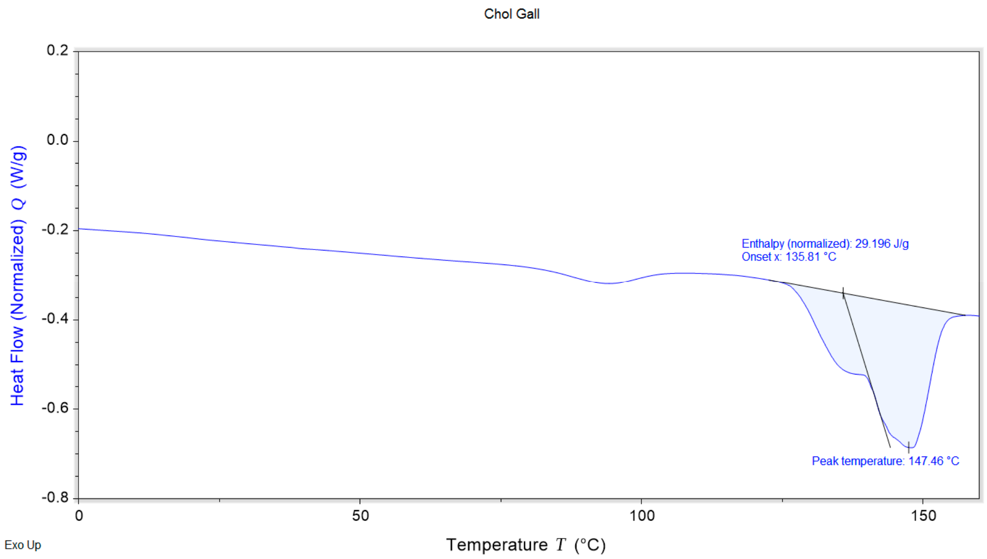

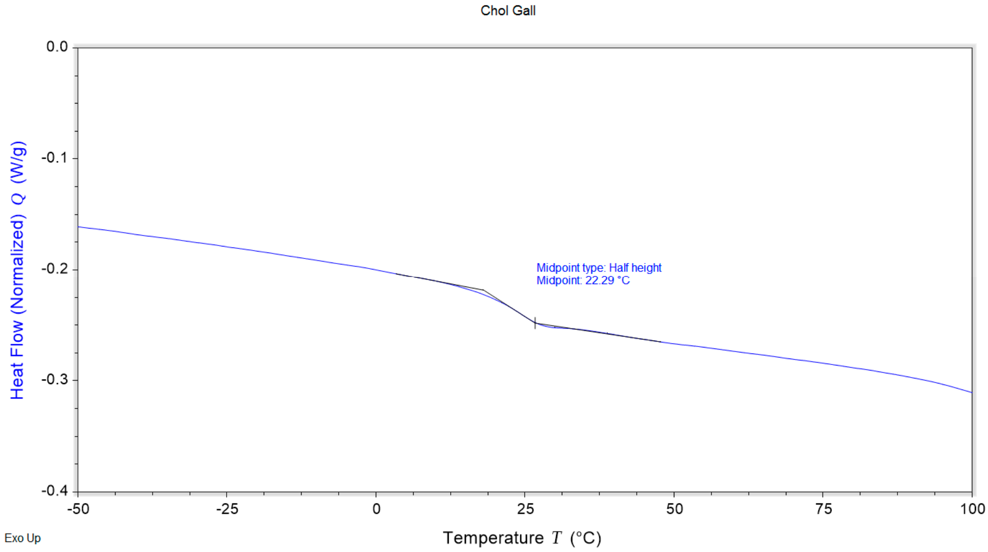

Figure S26 DSC curves of 1st and 2nd heating run of [Chol]Gal

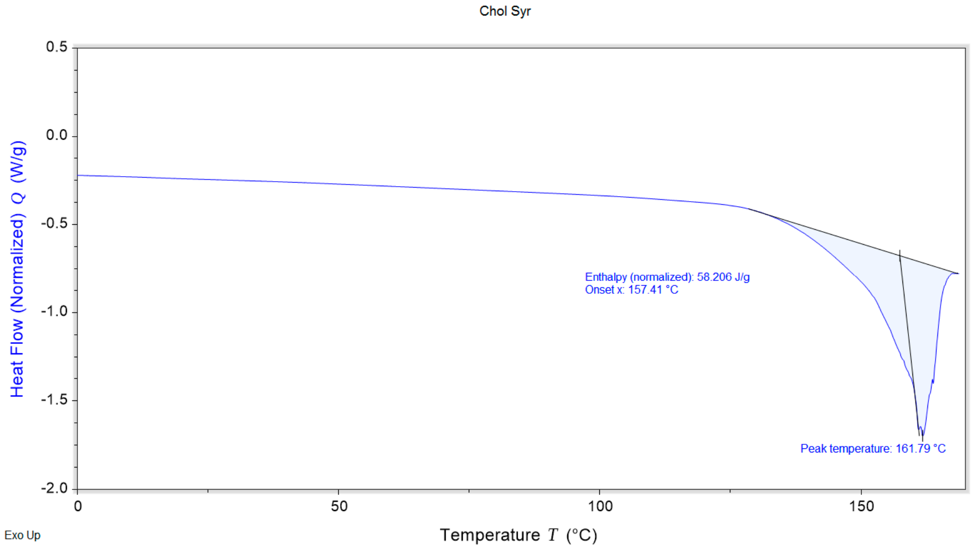

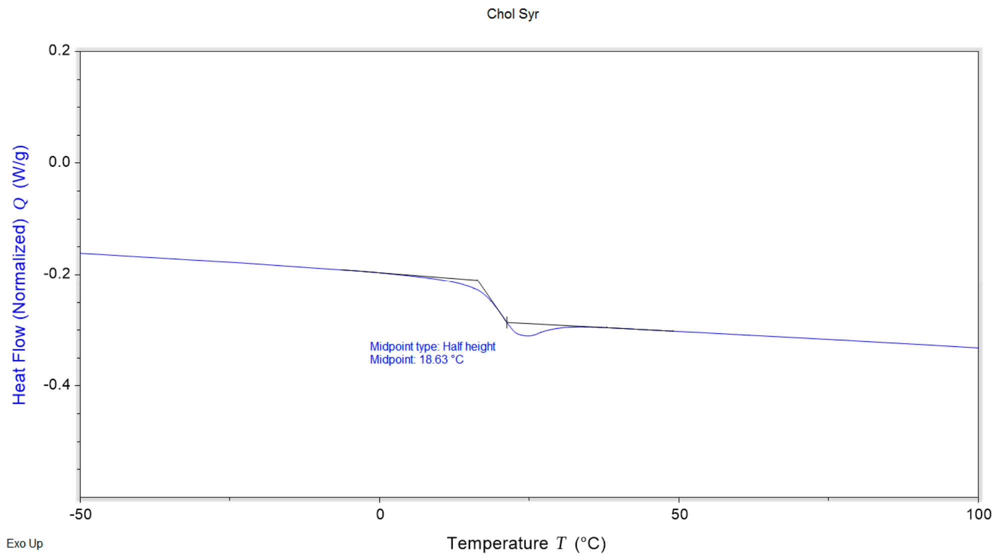

Figure S27 DSC curves of 1st and 2nd heating run of [Chol]Syr

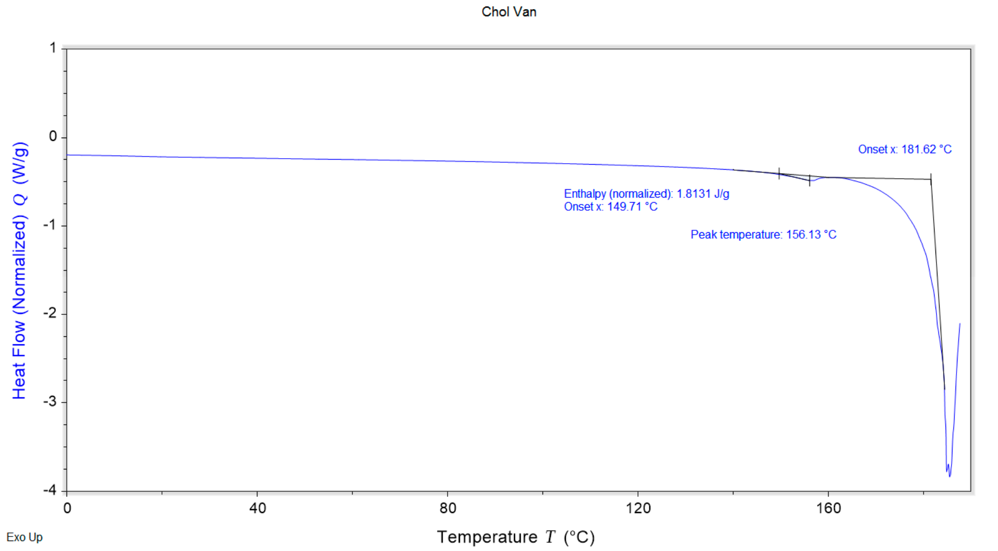

Figure S28 DSC curve of [Chol]Van

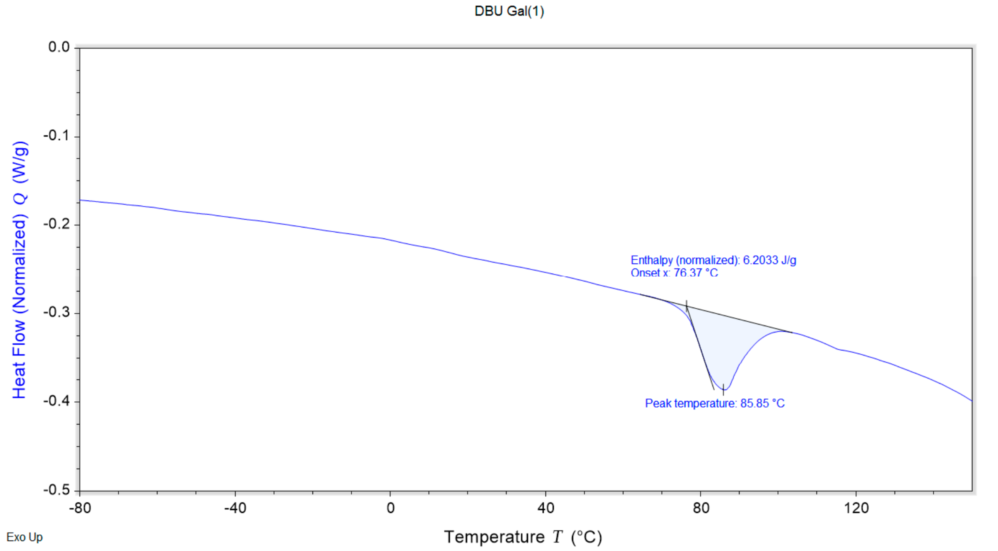

Figure S29 DSC curves of [DBU]Gal

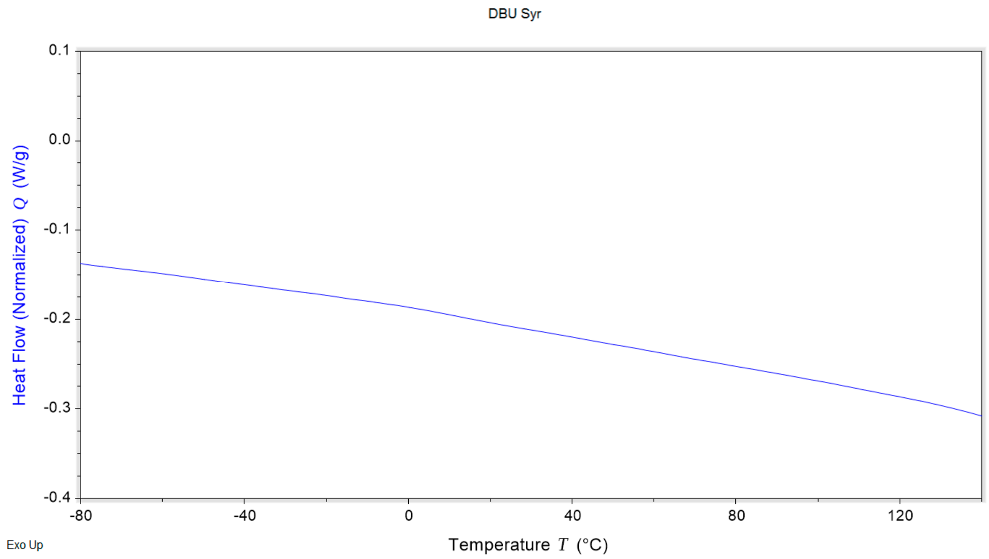

Figure S30 DSC curves of [DBU]Syr

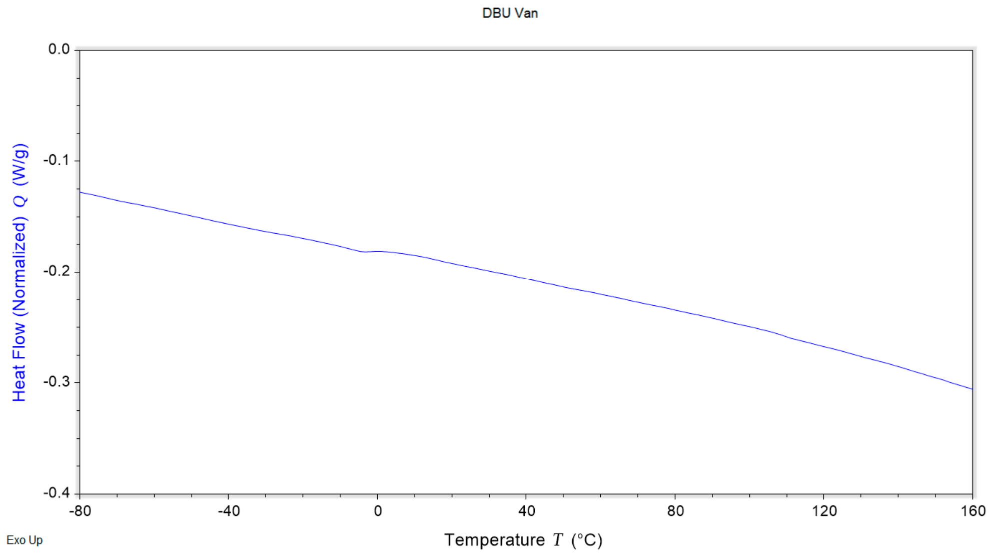

Figure S31 DSC curves of [DBU]Van
